# Advances in machine learning–enhanced microfluidic cell sorting

**DOI:** 10.1126/sciadv.aea6007

**Published:** 2025-12-19

**Authors:** Haodong Li, Jie Bai, Xiaxian Ma, Linwei Li, Yuanchao Liu, Xiaoyan Liu, Shaofei Shen, ChweeTeck Lim

**Affiliations:** ^1^ Shanxi Key Lab for Modernization of TCVM, College of Life Science, Shanxi Agricultural University, Taiyuan 030000, Shanxi, P. R. China.; ^2^ Department of Big Data and Intelligent Engineering, Shanxi Institute of Technology, Yangquan 045000, Shanxi, P. R. China.; ^3^ College of Information Science and Engineering, Shanxi Agricultural University, Taiyuan 030000, Shanxi, P. R. China.; ^4^ Institute for Health Innovation and Technology (iHealthtech), National University of Singapore, Singapore 117599, Singapore.; ^5^ Department of Biomedical Engineering, National University of Singapore, Singapore 117583, Singapore.; ^6^ Mechanobiology Institute, National University of Singapore, Singapore 117411, Singapore.

## Abstract

Cell sorting, essential for diagnostics and early intervention, has evolved from conventional methods to sophisticated microfluidic approaches. These miniaturized systems leverage precise hydrodynamic control, facilitating major advances in tumor cell isolation, single-cell analysis, and biomarker detection. However, the vast imaging data generated by these microfluidic techniques necessitate advanced computational methods. Machine learning, particularly computer vision and deep learning, now offers transformative capabilities for automated feature extraction, pattern recognition, and real-time classification, enhancing sorting accuracy, accelerating diagnostics, and informing clinical decisions. This review synthesizes the convergence of microfluidics and machine intelligence, examining their synergistic roles in flow-field optimization, cellular classification, and error correction. While highlighting breakthroughs in diagnostic sensitivity and analytical throughput, we critically address challenges including model generalizability and hardware-software integration. Last, we provide an outlook on multimodal data fusion and the development of on-chip intelligent systems, proposing a roadmap for advancing precision medicine through embedded, adaptive biosensing platforms.

## Teaser

Machine learning meets microfluidics as AI-driven cell sorting boosts diagnostic speed, accuracy, and precision medicine.

## INTRODUCTION

Cell sorting, as a pivotal technology for cellular characterization and specific subpopulation isolation, plays an irreplaceable role in early tumor screening (*1*, *2*), pathological mechanism investigation, and related fields. Traditional methods such as flow cytometry (FCM) and immunomagnetic bead sorting (*3*, *4*), while establishing foundational paradigms, are constrained by inherent limitations including operational complexity, throughput restrictions, and purity variability. Groundbreaking advances in microfluidic technology are reshaping the landscape of cell sorting: By developing dual-path sorting systems—active [relying on external field modulation via light (*5*), electricity (*6*), or magnetism (*7*)] and passive [based on channel geometry/fluid dynamics optimization (*8*)]—its miniaturized chip architectures, high-throughput processing capabilities, and precise sorting mechanisms have notably enhanced technical efficacy from tumor cell capture to single-cell analysis (*9*).

In the evolution of microfluidic sorting technologies, the intelligent advancement of detection and analytical systems has emerged as a pivotal breakthrough to address precision bottlenecks. Machine learning (ML), serving as the central engine for automated decision-making, is redefining biotechnological applications through data-driven pattern recognition and error-optimization algorithms (*10*). In drug development, its synergy with digital microfluidics accelerates compound screening (*11*); within droplet microfluidic systems, intelligent algorithms not only optimize droplet generation parameters (*12*) but also empower single-cell lipidomics analysis and cancer diagnostics. Notably, ML has achieved multidimensional breakthroughs in microfluidic chip design and fluid modeling, spanning emulsion size prediction to flow behavior analysis (*12*, *13*). Recent reviews on the integration of ML and microfluidics span a broad range of applications, including cell classification, drug screening, and biomarker detection. These articles summarize the use of ML across microfluidic cell analysis, biomarker detection, and droplet control, covering methods from classical algorithms (e.g., support vector machines) to deep convolutional networks, and highlight the potential of intelligent microfluidic systems to enhance throughput and automation (*14*). Other reviews center on characterization based on specific cellular biophysical properties—optical, mechanical, and electrical—providing systematic assessments of techniques for cell imaging, deformation analysis, and impedance measurement, along with their biomedical applications (*15*). Additional works emphasize the design automation of microfluidic devices or biosensing applications (*16*) or focus on single-cell analysis and specific sorting modalities (*17*).

**Fig. 1.**
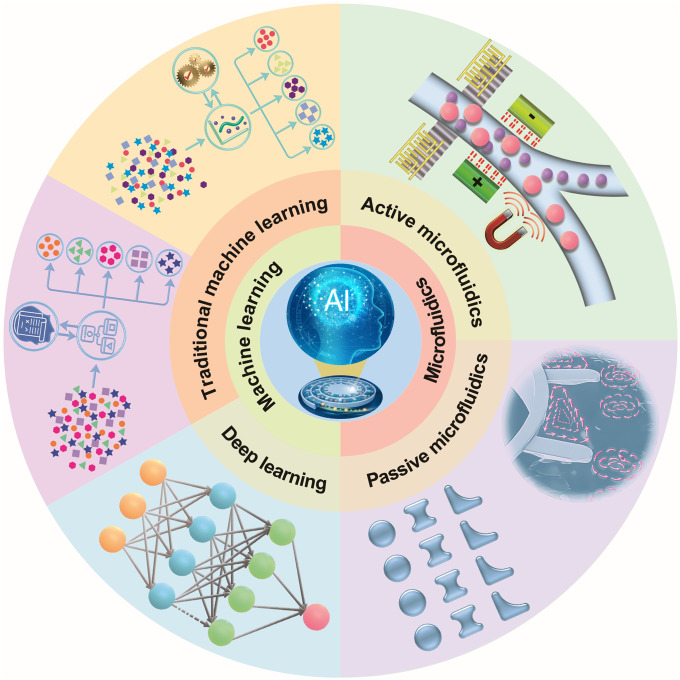
Schematic summary of advances in both active and passive microfluidic sorting technologies and integration of conventional ML and modern DL for intelligent cell sorting, illustrating the theme of “Advances in machine learning–enhanced microfluidic cell sorting.”

Despite this progress, a focused, systematic review of ML [especially deep learning (DL)] applied to microfluidic cell sorting is still lacking. Here, we synthesize advances in both active and passive microfluidic sorting technologies and delineate how conventional ML and modern DL have been integrated into these platforms (Fig. 1). By comparing and consolidating the prior literature, we clarify current capabilities, identify technical challenges, and outline emerging opportunities, providing a resource to guide the development of microfluidic cell sorting in the era of artificial intelligence (AI).

Building on the scholarly foundation, this article proposes a tripartite analytical framework—“technical principles, algorithmic integration, and application expansion.” First, we deconstruct the technological evolution of active/passive sorting mechanisms, highlighting the technical superiority of passive sorting as the mainstream approach. Second, a two-tier ML taxonomy is developed to comparatively analyze feature extraction capabilities between traditional supervised learning and modern DL architectures while elucidating their differential impacts on classification optimization. Last, by envisioning forward-looking directions such as on-chip intelligent systems and multimodal data fusion, the interdisciplinary roadmap for applying ML-empowered microfluidic sorting technologies to precision medicine and personalized therapeutics is provided.

## MICROFLUIDIC CELL SORTING TECHNOLOGY

Driven by the synergistic advancement of micro/nanofabrication technologies and microfluidic dynamics theory, microfluidic cell sorting has evolved into a dual-track technological framework encompassing “active-passive” strategies (Table 1). Active sorting achieves targeted capture through precise external field modulation, while passive sorting relies on microstructure-induced flow characteristics for label-free separation, creating complementary advantages in diagnostic sensitivity and throughput efficiency. Characterized by miniaturized integration, high-throughput processing, and cost-effective fabrication, this technology demonstrates a unique clinical value in scenarios such as circulating tumor cell enrichment and single-cell sequencing sample preparation. The integration of computer-aided design and multiphysics simulation technologies is propelling the evolution of sorting architectures from empirical trial-and-error approaches to intelligent optimization paradigms, establishing a transformative technological platform for precision medicine.

**Table 1.** Active/passive microfluidic cell sorting.

Category	Technique	Sample to separate	Efficiency	Throughput	Reference
Active microfluidics	MACS	*Vibrio parahaemolyticus*	–	40 min	(*21*)
	MACS	Circulating tumor-reactive lymphocytes	73.3%	4–24 ml/hour	(*2*)
	MACS	CTCs	85.1 ± 3.2%	60 μl/min	(*22*)
	DEP	A375, SK-MEL-1/2/28	99%	–	(*25*)
	DEP	PC3	–	3 μl/min	(*26*)
	Acoustofluidics	K-562	98%	Flow rate split ratio of 30%	(*44*)
	Acoustofluidics	MCF-7, HEK-293T	99.2%	1187 events/s	(*45*)
	Acoustofluidics	NK92, K562	>95.0%	1500 events/s	(*46*)
	Optical tweezers	MCF-7, Jurkat T	95.7%	–	(*52*)
	Optical tweezers	THP-1, yeast	>70%	–	(*53*)
Passive microfluidics	DLD	HGC-27	>80%	100 μl/min	(*60*)
	DLD	RBCs	95.3%	–	(*57*)
	DLD	MSCs	43.9 ± 6.3%	2.5 ml/20 ± 5 min	(*61*)
	DLD	CTCs	>96%	1 ml/min	(*63*)
	Inertial microfluidics	Receptor-T cell	70%	1 ml/min	(*90*)
	Inertial microfluidics	T cells	>80%	3.5 ml/min	(*91*)
	Inertial microfluidics	CHO cells	>97%	10 ml/min	(*92*)
	Inertial microfluidics	MCF-7, MDA-MB-231, A549	95%	240 μl/min	(*93*)
	Inertial microfluidics	*Haematococcus pluvialis*	99%	1–100 μl/min	(*94*)
	Viscoelastic microfluidics	WBCs, RBCs, PLTs	>97%	40 μl/hour	(*109*)
	Viscoelastic microfluidics	K562, HL60	–	0.05 ml/min	(*110*)
	Viscoelastic microfluidics	RBCs, WBCs, platelets	>80%	1 ml/min	(*111*)
	Viscoelastic microfluidics	MDA-MB-231	96.49%	3 μl/min	(*112*)

## ACTIVE MICROFLUIDIC CELL SORTING TECHNOLOGIES

**Fig. 2.**
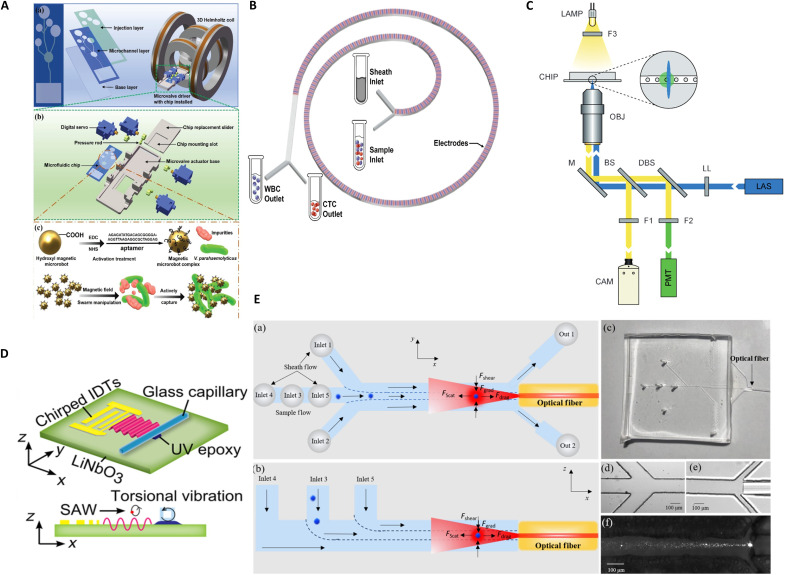
Active cell sorting. (**A**) A capillary-based microfluidic chip integrates immunomagnetic beads, with structural designs optimized for bead preparation and target cell capture. Image credits: Reprinted from (*21*), with the permission of AIP Publishing. (**B**) For cells with overlapping dimensions, a DEP-inertial microfluidic device achieves size-based separation through combined DEP and inertial focusing mechanisms. Image credits: Reused with permission from (*24*) (CC BY-NC-ND 4.0; https://creativecommons.org/licenses/by-nc-nd/4.0/). (**C**) Fluorescence-activated dielectrophoretic sorting of single-cell microfluidic droplets. Image credits: Reused with permission from (*31*). Used with permission of Royal Society of Chemistry from (*31*); permission conveyed through Copyright Clearance Center Inc. (**D**) Acoustic microcavity platform for enrichment of submicrometer/nanoscale particles via synergistic acoustic radiation force and single-vortex streaming-induced viscous effects. Image credits: Reprinted in part with permission from (*42*). Copyright 2017 American Chemical Society. (**E**) Optofluidic dynamic tweezers offer precise manipulation of particles through light-driven hydrodynamic fields, demonstrating versatility in noncontact sorting applications. Image credits: Reused with permission from (*53*) (CC BY-NC 3.0; https://creativecommons.org/licenses/by-nc/3.0/).

Active microfluidic cell sorting enables trajectory programming of cells through externally field-mediated manipulation, with its core lying in establishing coupled “physical field-cell property” interaction mechanisms. As fundamental field modulation elements, multimodal energy fields—including magnetic force, dielectrophoretic force, acoustic radiation force, and optical tweezers (OTs)—allow precise subpopulation separation based on cellular physical fingerprints such as size, dielectric properties, and magnetic susceptibility. This section systematically analyzes the evolutionary trajectory and technical characteristics of active sorting technologies: First, elucidating the physical operating principles of four mainstream approaches—magnetic-activated cell sorting (MACS), dielectrophoretic sorting [dielectrophoresis (DEP)], acoustofluidic sorting, and OT-based sorting. Subsequently, through representative case studies, we unveil their technological progression patterns (Fig. 2). Last, we construct a triaxial evaluation framework (“principle-performance-limitation”) to critically examine common technical bottlenecks, including field cross-talk, biocompatibility constraints, and throughput-resolution trade-offs, thereby providing theoretical guidance for optimizing cross-scale cell manipulation systems.

### Magnetic-activated cell sorting

MACS establishes a biophysical sorting system based on antigen-antibody specificity: Superparamagnetic nanoparticles serve as carriers, forming immunomagnetic bead complexes through covalent conjugation of surface-specific antibodies. When incubated with mixed-cell suspensions, these complexes bind to target cells via epitope-antibody interactions, generating bead-cell conjugates. Under applied gradient magnetic fields, labeled cells are retained in the separation zone because of magnetic hysteresis, while unlabeled cells are eluted via laminar flow, achieving high-purity target cell isolation (*18*). The technology comprises two labeling strategies: (i) Positive selection directly captures target cells (forming bead-antibody-target ternary complexes) (*19*); (ii) negative selection enriches targets indirectly by labeling nontarget cells (*20*). While positive selection offers operational simplicity, it risks aberrant signaling from membrane receptor cross-linking. Conversely, negative selection preserves native cell states but suffers from reduced efficiency in low-abundance samples because of nonspecific adsorption and impurity retention.

Zhang *et al.* (*21*) developed a capillary force–driven microfluidic system that automates magnetic bead manipulation through gas-liquid interfacial self-assembly, notably enhancing detection automation, efficiency, and reproducibility (Fig. 2A). Wang *et al.* (*2*) established a MACS platform, providing a nonsurgical alternative for isolating tumor-reactive lymphocytes from peripheral blood. This approach highlights the potential of passive immunotherapy using circulating tumor-reactive lymphocytes, overcoming the clinical challenge of procuring fresh tumor specimens. Further advancing this field, Wang *et al.* (*22*) designed a hybrid microfluidic chip integrating deterministic lateral displacement (DLD) with MACS, achieving an 85.1 ± 3.2% circulating tumor cell capture efficiency at 60 ml/min flow rates alongside a 99.9% leukocyte clearance.

Despite ISO 13485 certification, the clinical translation of magnetic-activated sorting faces three persistent barriers: (i) high manufacturing costs of magnetic nanoparticles, notably exceeding traditional centrifugation methods; (ii) recovery rate variability in rare cell populations (<0.01%) because of signal-to-noise ratio attenuation; (iii) nonspecific adsorption caused by ligand dissociation from magnetic bead surfaces. Emerging strategies to overcome these limitations include developing bioinspired aptamer-functionalized magnetic beads to minimize nonspecific binding, designing microfluidic fluidized-bed systems to enhance rare cell capture efficiency, and integrating magnetophoretic-dielectrophoretic hybrid sorting to achieve a single-cell resolution. Theoretically, advancing collective magnetic behavior studies and establishing synergistic models of magnetophoretic effects with convective entrapment will guide next-generation multiphysics-coupled sorting devices, addressing scalability and precision trade-offs inherent in current platforms.

### Dielectrophoretic microfluidic (DEP) separation

Microfluidic DEP achieves particle manipulation via polarization effects induced by nonuniform electric fields, with its mechanism rooted in the complex permittivity contrast between particles and the surrounding medium (*23*). When biological molecules or cells are subjected to nonuniform electric fields, interactions between induced/intrinsic dipole moments generate a net dielectrophoretic force. This force, independent of particle charge, is governed by the coupled relationship among medium-particle electrical properties, geometric morphology, and field frequency. By precisely modulating field frequency, selective manipulation of specific cell subpopulations [e.g., circulating tumor cells (CTCs) versus white blood cells (WBCs)] can be achieved, establishing a frequency-dependent “field signature” separation system (Fig. 2B) (*24*).

Chen *et al.* (*25*) developed a DEP-based wireless bipolar electrode platform for the highly efficient separation of circulating melanoma cells from blood. Under optimized conditions at 50 kHz, the platform achieved a separation efficiency of 99% with extremely low WBC contamination (1:10^4^ to 10^8^). The current throughput allows processing of 10 to 15 μl of whole blood (~1.2 × 10^6^ cells) within 100 min. Its parallel channel design offers strong scalability, demonstrating potential to meet clinical throughput requirements. Complementing this advancement, Farasat *et al.* (*26*) developed a DEP-based approach using replaceable polydimethylsiloxane porous membranes for CTC capture. The applied DEP forces immobilized CTCs within membrane pores, facilitating on-chip functional analyses while preserving cell viability. This platform demonstrated exceptional sensitivity in detecting rare cells (<0.01% abundance) within large cellular populations, addressing a critical challenge in liquid biopsy applications.

Notably, the synergistic coupling of DEP with optical fluorescence labeling establishes a dual-mode identification system for droplet microfluidic sorting. This approach generates water-in-oil emulsion droplets (50- to 200-μm diameter) encapsulating single cells, where fluorescent probe–labeled targets are selectively deflected via DEP upon exceeding predefined fluorescence intensity thresholds, achieving a 98% sorting accuracy (*27*). The technology has been expanded through fluorescent encoding strategies (e.g., fluorescein isothiocyanate/phycoerythrin dual labeling) to applications including high-throughput screening of filamentous fungi (*28*), directed enzyme evolution (*29*), and antibody affinity sorting (*30*). The fluorescence-activated droplet sorting system developed by Baret *et al.* (Fig. 2C) (*31*) exemplifies this paradigm, enabling DEP-mediated sorting at 2000 droplets/s while combining the throughput of microtiter plates with the precision of fluorescence-activated cell sorting. By automating droplet deflection triggered by fluorescence signals, this method replaces manual operations and provides high-purity samples for single-cell omics (*32*). While ML algorithms have introduced intelligent decision-making by translating expert knowledge into quantifiable classification models, practical implementation faces two critical constraints: (i) Droplet sorting efficacy relies on sufficient surface charge or physicochemical heterogeneity to generate actuation forces (*33*), and (ii) dc electric field–induced membrane potential perturbations may compromise cellular viability (*34*). These limitations underscore the need for advanced DEP control strategies balancing sorting efficiency with biospecimen integrity.

DEP offers notable advantages for cell sorting by enabling label-free separation based on intrinsic biophysical properties rather than surface markers, making it particularly suitable for isolating rare or heterogeneous populations such as circulating melanoma cells, which often lack specific antigens. The technique uses microfabricated electrodes—including wireless bipolar or interdigitated structures—to generate highly controllable nonuniform electric fields that selectively capture and isolate individual cells under laminar flow, thereby facilitating subsequent single-cell analysis. However, DEP applications face challenges because of complex and incompletely decoupled multiphysical mechanisms: Nonlinear interactions among factors such as medium permittivity, field frequency, and cell size complicate performance prediction, while variability in the dielectric properties of biological samples (e.g., broader response distributions in peripheral blood mononuclear cells from advanced-stage patients) further impedes protocol standardization. Additional practical limitations include dependence on low-conductivity buffers that may affect cell physiology and risks from Joule heating under high-voltage operation that could compromise cell integrity. Future efforts should prioritize developing multiscale models to improve predictive accuracy and advancing biocompatible electrode materials to minimize thermal effects, thereby enhancing the reliability and scalability of DEP for clinical applications such as CTC sorting.

### Acoustofluidic separation

Acoustofluidic technology enables label-free sorting of biological nanoparticles through acoustic wave-matter interactions, with separation mechanisms relying on intrinsic physical property differentials such as particle size, density, and compressibility (*35*, *36*). High separation resolution is ensured by the low-Reynolds-number laminar flow within microfluidic channels, surpassing conventional bulk fluidic systems in precision. This approach achieves on-chip target enrichment without requiring high temperatures, shear stress, or chemical modification. However, current implementations face industrialization barriers as a result of the bulky architecture of piezoelectric actuation modules and the substantial costs associated with specialized electronic instrumentation. Emerging innovations aim to miniaturize transducer components and integrate cost-effective driving circuits to bridge laboratory-scale prototypes to clinical-grade platforms (*37*).

Acoustofluidic sorting operates through two principal modalities: bulk acoustic wave, driven by bulk piezoelectric transducers, and surface acoustic wave (SAW), generated via interdigitated transducers to establish high-frequency (megahertz-range) surface waves along channel sidewalls (*38*). SAW has emerged as the preferred microfluidic actuation strategy because of its miniaturized IDT arrays and low-power operation, directly advancing the development of on-chip acoustofluidic systems (*39*). Its contactless, biocompatible nature and excellent spatiotemporal resolution have established it as a powerful platform for point-of-care diagnostics and fundamental biological research (*40*). SAW further bifurcates into traveling SAW (TSAW) and standing SAW (SSAW) configurations: TSAW induces acoustic streaming via unilateral IDT excitation, while SSAW creates pressure node arrays through bilateral IDT interference (*41*).

In devices based on SAWs (SSAW), the primary mechanism for particle manipulation is dominated by the acoustic radiation force (*18*). This force arises from the scattering of incident acoustic waves at the particle-fluid interface, and its magnitude and direction are determined by the particle’s acoustic contrast factor (Φ)—a dimensionless parameter defined by the ratio of particle to medium density (ρ_p/ρ_m) and compressibility (β_p/β_m). When Φ is positive (Φ > 0), particles are driven toward pressure nodes; when negative (Φ < 0), they migrate toward pressure antinodes. It is important to distinguish acoustic streaming–based separation from active sorting: The former is a continuous, passive process that leverages inherent differences in Φ to separate heterogeneous populations into distinct fluid streams, whereas the latter involves real-time detection of specific targets (e.g., fluorescently labeled cells) followed by triggered deflection into collection outlets. A complex interplay exists between the primary radiation force and secondary acoustic streaming effects—while radiation forces dominate transverse particle positioning, streaming-induced viscous drag can oppose or enhance this motion, particularly near channel boundaries or with nanoscale objects, notably influencing separation resolution and sorting purity.

Acoustofluidic systems enable label-free precise manipulation of nanoparticles through acoustic field-microfluidic coupling. When acoustic waves interact with particles, material properties (density and compressibility) and size differences generate differential acoustic radiation forces, facilitating capture, focusing, and spatial patterning. Mao *et al.* (*42*) developed a low-power acoustofluidic chip that establishes a single-vortex acoustic field within a glass capillary under a 5-V driving voltage. Through the synergistic interplay between acoustic radiation and viscous drag forces, this device achieves streamline focusing of 80- to 500-nm silica/polystyrene particles (Fig. 2D). Furthermore, the platform successfully enriches bovine serum albumin-biotin–coated nanoparticles in minute sample volumes, enhancing immunoassay signals via efficient capture of Alexa 488–labeled streptavidin. Emerging acoustic holography techniques (*43*) extend manipulation versatility by programming acoustic phase distributions to generate arbitrary patterned fields.

The focusing behavior of particles or cells under an acoustic field provides a robust foundation for highly efficient sorting based on physical properties. By precisely controlling the acoustic field, different cell types can be steadily focused into specific streamlines or regions, notably enhancing the resolution, purity, and throughput of target cell isolation from complex samples. Soller *et al.* (*44*) first reported that cells in whole blood can undergo self-organization under tightly focused acoustic fields. Using acoustic radiation forces, red blood cells were concentrated at the channel center to form a heterogeneous acoustic medium, achieving cancer cell enrichment ratios of 14- to 43-fold at a 70% split ratio and recovery rates >98% at a 30% split ratio and 100 μl/min flow rate, maintaining >95% even at 300 μl/min. Zhong *et al.* (*45*) demonstrated high-performance acoustic sorting in whole blood based on cell-medium acoustic interaction. They developed a high-throughput, high-purity acoustofluidic droplet sorter using a single-phase focused transducer, which achieved sorting of more than 1187 droplets per second with an input voltage below 20 Vpp (peak-to-peak voltage), a purity of 99.2%, and a cell viability of 93.5%. Further advancing the technology, Zhong *et al.* (*46*) developed a modular acoustofluidic platform named CIAMAP, capable of processing up to 1500 events per second with more than 95% accuracy, while supporting real-time dynamic analysis and multiomics validation, greatly improving the efficiency and reliability of cellular immunology research.

Acoustofluidic cell sorting systems exhibit high biocompatibility, a key advantage for biomedical applications, owing to the effective management of thermal effects and cavitation risks. Although piezoelectric transducers generate heat during operation, integrated thermal management strategies—such as polymer-based heat-dissipating layers—effectively maintain temperatures within biologically acceptable limits. Under standard high-frequency operation and optimized acoustic intensity, cavitation effects caused by microbubble collapse can be notably suppressed. Crucially, standing-wave acoustophoresis provides inherent cell protection: Cells with a positive acoustic contrast factor (Φ > 0) are focused toward pressure nodes, while cavitation-prone microbubbles (Φ < 0) migrate toward pressure antinodes, thereby spatially isolating cells from potential damage. However, the technology still faces several challenges, including sensitivity to sample hematocrit, reduced sorting efficiency at high flow rates, and reliance on polydimethylsiloxane materials—factors that currently hinder large-scale manufacturing and clinical translation. Future efforts should focus on developing robust acoustic control systems, integrating real-time sensing technologies, and adopting modular plastic-chip designs to advance high-throughput, clinically viable cell sorting platforms.

### OT microfluidic sorting

OTs, a precision technology for microscale particle manipulation based on radiation pressure effects arising from light-matter interactions, use a single tightly focused laser beam to generate three-dimensional (3D) gradient potential wells (*47*). This enables noncontact trapping and precise control of micrometer-scale particles, hence termed “single-beam gradient optical traps.” The foundational work in this field dates to the theoretical framework of optical manipulation proposed by Ashkin in 1970 (*48*). A groundbreaking advancement emerged in 1987 when Ashkin and Dziedzic (*49*) demonstrated that a single strongly focused laser beam could establish stable optical trapping systems, laying the theoretical groundwork for modern OT technologies.

OT systems comprise two functional modules: optical trapping and optical manipulation. The trapping mechanism originates from momentum transfer effects during light-matter interactions. When a laser beam is tightly focused through a high-numerical-aperture objective, it generates an intense gradient field near the focal region. The resultant optical forces can be decomposed into two vector components: scattering force (Fs) and gradient force (Fg). The scattering force, arising from photon reflection and absorption, propels particles along the beam’s propagation axis, while the gradient force, derived from the transverse intensity gradient of the Gaussian beam, confines particles to regions of maximum light intensity. By dynamically balancing these forces, 3D stable trapping at the focal point is achieved.

Optical manipulation is realized through spatiotemporal light field modulation, primarily via passive and active modes. Passive manipulation involves precisely translating the sample stage to alter the relative positions between trapped particles and the microfluidic environment. In contrast, active manipulation uses components such as acousto-optic deflectors to directly steer the trapping beam’s trajectory. This noncontact control paradigm demonstrates unique advantages in microfluidic systems, offering critical technical capabilities for biomedical applications ranging from cell sorting to single-molecule manipulation.

OTs, capable of manipulating targets from tens of nanometers to hundreds of micrometers, are uniquely suited for biological systems—spanning single cells (1 to 100 μm), organelles (0.5 to 5 μm), and biomolecules (10 to 100 nm)—enabling groundbreaking applications in single-cell analysis and molecular interaction studies (*50*, *51*). Recent advancements bifurcate into two transformative directions: (i) multimodal integration, exemplified by Zheng *et al.*’s fluorescence-activated OT sorting (FACS-OT) system (*52*), which combines microfluidics with fluorescent nanoprobes to achieve a 95.7% deflection efficiency in CTC isolation, and (ii) previously unidentified actuation mechanisms, such as Vasantham *et al.*’s optohydrodynamic fiber tweezers (*53*), which synergize 3D hydrodynamic focusing with photonic force fields for high-throughput particle manipulation in microchannels (Fig. 2E). The device achieves a sorting efficiency exceeding 70% at flow velocities up to 1000 μm s^−1^. By adjusting optical power and flow rate, it enables precise capture and long-range manipulation of particles and cells with diverse sizes, shapes, and material compositions within the channel. These innovations exemplify the shift toward label-free specificity, dynamic programmability, and cross-scale compatibility, positioning OTs as indispensable tools for probing cellular dynamics and molecular interactions in native microenvironments.

Despite its advantages in precise manipulation, OTs exhibit notable limitations in cell sorting applications. First, high-intensity laser exposure poses a risk of photodamage to live cells—especially under prolonged or high-power operation—which restricts their use in sensitive biological samples. Second, although optofluidic systems enable noncontact single-cell sorting, their processing throughput remains substantially lower than that of conventional FCM, making them unsuitable for high-throughput scenarios. Furthermore, system integration is highly complex, requiring high-numerical-aperture objectives, customized microfluidic chips, and real-time fluorescence detection and feedback control systems. This leads to high costs and specialized operational demands, hindering broader adoption. While recent advances have improved throughput and manipulation range, further optimization of system efficiency and reduction in complexity are essential to advance OTs in cell sorting.

## PASSIVE MICROFLUIDIC CELL SORTING TECHNOLOGIES

This section systematically reviews mainstream passive microfluidic sorting strategies, focusing on three core methodologies: DLD, inertial microfluidics, and viscoelastic microfluidics. Techniques such as microfiltration, sedimentation, adhesion-based sorting, and hydrodynamic focusing are excluded on the basis of technological maturity and application scope. For each selected approach, we first dissect the mathematical models governing their separation mechanisms and flow-field characteristics. Subsequently, we trace the technological evolution of critical parameter optimization pathways. Last, representative application cases are analyzed to elucidate their efficacy and throughput profiles for isolating specific cell populations, including CTCs, leukocytes, and stem cells.

### Deterministic lateral displacement

**Fig. 3.**
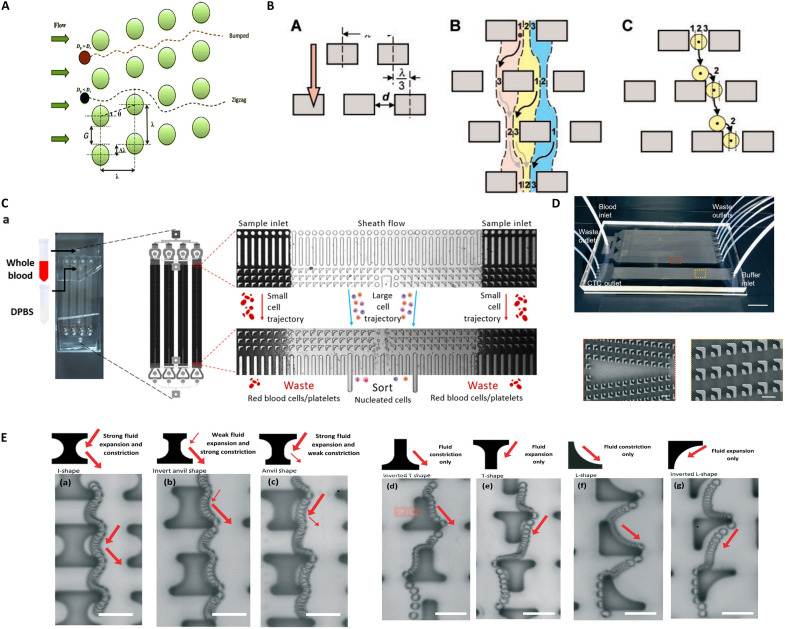
DLD. (**A**) Variations in pillar configurations within DLD devices. Image credits: Reprinted from (*54*), with the permission of AIP Publishing. (**B**) Schematic of the DLD principle, featuring a periodic array of micrometer-sized pillars. Image credits: Reproduced/modified from (*55*). (**C**) Structure of a parallel inverted L-shaped DLD chip for nucleated cell sorting from whole blood, with performance characterization: Nucleated cells are collected in the “sorted” stream, while the waste stream comprises red blood cells and platelets. Image credits: Reused with permission from (*61*) (CC BY-NC 3.0; https://creativecommons.org/licenses/by-nc/3.0/). (**D**) Filter-based highly integrated cascaded chip (CFD-Chip) for enhanced separation efficiency. Image credits: Reused with permission from (*63*). Used with permission of Royal Society of Chemistry from (*63*); permission conveyed through Copyright Clearance Center, Inc. (**E**) Sequential positions and fluid flow variations of identical microspheres in distinct pillar geometries: (a) I-shaped, (b) anvil-shaped, (c) inverted anvil-shaped, (d) inverted T-shaped, (e) double inverted T-shaped, (f) L-shaped, and (g) inverted L-shaped. Image credits: Reused with permission from (*57*). Used with permission of Royal Society of Chemistry from (*57*); permission conveyed through Copyright Clearance Center Inc.

DLD is a microfluidic sorting technology that exploits differences in physical properties of particles under laminar flow, with separation parameters including cell size, morphology (e.g., sphericity), and deformability (Fig. 3A) (*54*). Pioneered by Huang *et al.* (*55*) and later systematized by Wu and Hjort (*56*), DLD devices feature periodic micropillar arrays with horizontally offset rows at specific ratios, forming asymmetric flow networks (Fig. 3B). The DLD system uses a periodic array of micrometer-scale pillars with uniform spacing. Each successive row of pillars is offset horizontally by a specific distance. Under low-Reynolds-number laminar flow, particles move along size-dependent paths. Particles smaller than a critical size follow a zigzag motion through the pillars. These subcritical particles largely stay on their original streamlines. In contrast, particles larger than the critical size experience a systematic lateral shift. The pillar offset angle governs this lateral displacement. This behavior separates the particles into different trajectories. The system then directs each group to a separate outlet on the basis of its diameter. Engineers can tune the critical size threshold by optimizing geometric parameters. These parameters include the pillar gap, offset ratio, and channel height. Relying solely on hydrodynamic interactions, DLD performs label-free, high-resolution sorting. Its applications range from cell isolation and extracellular vesicle enrichment to synthetic particle fractionation (*57*).

DLD technology has achieved efficient sorting of bioparticles spanning microscale to nanoscale dimensions, including polystyrene beads (micrometer/nanosized), droplets, DNA, exosomes (*58*), blood cell subtypes, and CTCs (*59*). Zhao *et al.* (*60*) developed an integrated microfluidic device combining Dean flow fractionation (DFF) and DLD for the isolation of free gastric cancer cells from background cells. Operating at a flow rate of up to 100 μl/min, the chip achieved a recovery rate of 84.8% and a sorting purity of 72.4%. This platform offers a powerful label-free technology for the clinical diagnosis of peritoneal metastasis in gastric cancer. Ranjan *et al.* (*57*) designed “I-shaped” arrays to eliminate dead flow zones, achieving >90% separation efficiency for spherical/nonspherical particles. Tan Kwan Zen *et al.* (*61*) used inverted L-shaped pillars to enrich scalable mesenchymal stem cells (MSCs) (Fig. 3C), while Loutherback *et al.* used triangular pillars to enhance shear-mediated isolation of viable CTCs from blood (*62*). Building on this advancement, Liu *et al.* (*63*) proposed a “filter-DLD” concept via cascaded chips coupling size exclusion with inertial focusing, enabling single-step CTC/circulating hybrid cell sorting with >96% capture efficiency, 99.995% leukocyte depletion, and >98% cell viability (Fig. 3D). This platform simultaneously supports single-cell transcriptomic sequencing, establishing a high-throughput, high-fidelity solution for liquid biopsy in oncology.

Recent advancements in nanofabrication have propelled DLD technology into the realm of nanoscale precision. Wunsch *et al.* (*64*) pioneered true nanoscale DLD arrays with pillar gaps ranging from 25 to 235 nm, achieving unprecedented separation of nanoparticles (20 to 110 nm) and exosomes, thereby transcending traditional size limitations. Expanding on this, Gaillard *et al.* (*65*) developed targeted nano-DLD devices capable of isolating extracellular vesicles from cell cultures and blood samples, underscoring the technology’s potential in nanobiomedical analysis. These breakthroughs highlight DLD’s unique capability for size-selective nanoparticle manipulation with a sub–100 nm resolution. In addition, DLD systems are evolving beyond standalone sorting through modular integration. Castaño *et al.* (*66*) exemplify this trend with a DLD-MS–integrated platform combining DLD, microfluidic mixing, and magnetic separation (MS). This system isolates allergy-related basophils directly from whole blood within 10 min, achieving dual 95% purity and recovery rates. By cascading physical sorting (DLD) with magnetic labeling (MS), the platform bypasses cell prelabeling steps that compromise viability while ensuring uniform biointerface interactions via integrated mixers. The “sort-enrich-analyze” workflow streamlines basophil activation testing, offering standardized, minute-scale diagnostics for clinical allergy management.

DLD has evolved over two decades into a multiscale separation platform, enabled by synergistic optimization of post architectures (circular, triangular, I-shaped, and inverted-L configurations) and physical markers (size, morphology, and deformability) (Fig. 3E), achieving cellular to nanoscale target resolution with milliliter-per-minute throughput. Nevertheless, clinical implementation faces critical barriers: mandatory sample predilution to mitigate flow-field perturbations, separation threshold instability in viscous biofluids, inefficient submicrometer particle resolution, and cost-prohibitive nanoscale fabrication tolerances coupled with intricate fluid-structure interactions. In addition, cell sorting mainly faces the risk of clogging, high manufacturing complexity (especially 3D structures), and critical diameter (*D*_c_) limitations, which limit its performance and practicality in high-throughput applications. Future breakthroughs demand three priorities: dynamically reconfigurable post arrays for adaptive threshold control, modular chip architectures integrating separation with single-cell omics interfaces, and AI-enhanced fluid dynamics modeling to decode biological complexity. Through scalable manufacturing and system integration, DLD is poised to transition from laboratory innovation to a cornerstone technology for clinical diagnostics and precision medicine. First is the shift from static optimization to dynamic reconfiguration by using microfluidics, optofluidics, or electrowetting. These technologies can create column arrays that regulate the critical separation size (*D*_c_) in real time. This greatly enhances application flexibility. Second is moving from isolated sorting modules to fully integrated systems by designing standardized modular architectures. These should seamlessly combine DLD with downstream single-cell operations and multiomics analysis interfaces, creating a one-stop “sorting-to-analysis” solution. Third is the transition from physical intuition to AI prediction by using AI-enhanced fluid models to guide inverse chip structure design. Intelligent integration of complementary technologies is used to accelerate development for complex biological samples. Ultimately, with scalable manufacturing techniques such as nanoimprinting, DLD technology can transition from the laboratory to clinical practice, becoming a key enabler for precision medicine.

### Inertial microfluidics

**Fig. 4.**
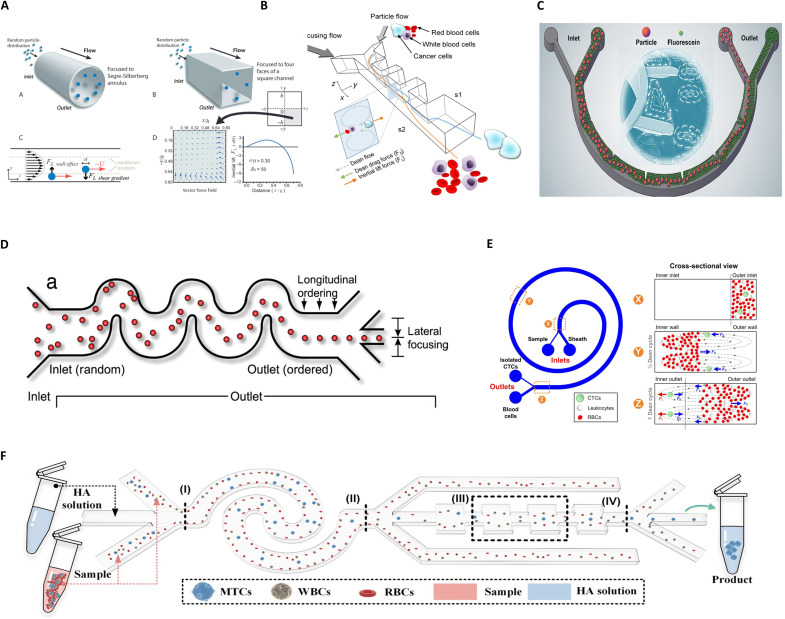
Inertial microfluidics. (**A**) Fundamental principles of inertial microfluidics, highlighting particle focusing and size-dependent migration. Image credits: Reused with permission from (*75*). Used with permission of Royal Society of Chemistry from (*75*); permission conveyed through Copyright Clearance Center Inc. (**B**) Inertial microfluidic separation of cancer cells from blood under low shear stress. Image credits: Reprinted in part with permission from (*72*). Copyright 2013 American Chemical Society. (**C**) High-throughput buffer exchange system using a semicircular microchannel with ordered micro-obstacles. Image credits: Reprinted in part with permission from (*74*). Copyright 2025 American Chemical Society. (**D**) Serpentine inertial microfluidic channel for enhanced particle focusing and separation efficiency. Image credits: Reused with permission from (*76*). Copyright (2007) National Academy of Sciences, US. (**E**) Schematic of Dean flow fractionation (DFF) for high-throughput isolation of CTCs. Image credits: Reused with permission from (*73*). (**F**) Cascaded elastic-inertial cell separation device for size-selective sorting of biological particles. Image credits: Reused with permission from (*93*) (CC BY-NC 3.0; https://creativecommons.org/licenses/by-nc/3.0/).

Inertial microfluidics leverages nonlinear hydrodynamic effects at intermediate Reynolds numbers to enable label-free, high-throughput manipulation of bioparticles (Fig. 4) (*67*–*70*) Its operational framework encompasses two synergistic modes: inertial focusing in straight channels (Fig. 4A) and secondary flow modulation in curved geometries (Fig. 4, B and C) (*71*–*74*). In straight microchannels, particles undergo lateral migration governed by competing shear-induced and wall-induced lift forces, stabilizing at equilibrium positions for rapid self-organization into ordered streams (*75*). Curvilinear architectures (spiral/serpentine) enhance control by generating Dean vortices through centrifugal pressure gradients, which couple transverse migration with inertial focusing to achieve size-based separation (Fig. 4, D and E) (*17*, *73*, *76*). These principles underpin diverse biomedical applications, including pathogen detection (*77*), CTC isolation (*78*, *79*), and integration with single-cell omics workflows (*80*). The technology’s label-free nature preserves cellular integrity, while its scalable throughput and compact designs align with point-of-care diagnostic requirements (*81*, *82*). By harmonizing inertial and secondary flow effects, inertial microfluidics bridges fundamental hydrodynamics with precision biomedicine, offering a versatile platform for next-generation cellular analysis (*83*).

Inertial microfluidics has emerged as a versatile platform for bioparticle manipulation, leveraging its inherent advantages of high throughput, operational simplicity, and minimal biomolecular interference (*84*). This technology enables diverse biomedical applications, including CTC isolation (*85*), single-cell droplet encapsulation (*86*), pathogen removal, exosome detection (*87*), and DNA focusing, offering a robust tool for label-free separation and analysis. Researchers are advancing channel geometry designs to enhance focusing precision and separation efficiency, while computational fluid dynamics simulations provide critical insights into the underlying hydrodynamic mechanisms (*88*, *89*). These efforts aim to optimize inertial migration effects and secondary flow interactions, bridging fundamental fluid dynamics with practical biomedical workflows. The technology’s scalability and compatibility with downstream analytical modalities position it as a cornerstone for next-generation diagnostic and therapeutic platforms.

Recent studies underscore the expanding utility of inertial microfluidics in therapeutic development and biomanufacturing. Elsemary *et al.* (*90*) leveraged inertial focusing to enrich T lymphocytes from leukemia patient blood (B cell acute lymphoblastic leukemia/chronic lymphocytic leukemia), streamlining chimeric antigen receptor T cell production. Jeon *et al.* (*91*) used multidimensional double-spiral architectures to isolate activated, transduced T cells on the basis of size differences, enhancing chimeric antigen receptor T cell manufacturing efficiency. In bioprocessing, Kwon *et al.* (*92*) demonstrated selective removal of oversized CHO cell clusters from suspension cultures using single-spiral inertial microfluidics, optimizing cell viability and protein yield. The integration of elastic and inertial effects has emerged as a promising paradigm for complex biological samples. Ni *et al.* (*93*) developed a cascaded elastic-inertial cell separation device to isolate malignant tumor cells from malignant pleural/peritoneal effusions, notably improving diagnostic sensitivity (Fig. 4F). Concurrently, Yan *et al.* (*94*) pioneered a tunable elastic-inertial microchannel for size-based separation of *Haematococcus pluvialis* cells, achieving adjustable separation thresholds through optofluidic modulation. These advancements highlight the adaptability of inertial microfluidics in addressing heterogeneous cellular populations across therapeutic and industrial bioprocessing workflows.

Our group pioneered a microstructure-enhanced inertial microfluidic platform that addresses critical bottlenecks in conventional systems through four synergistic advantages: (i) streamlined fabrication via single-layer soft lithography and large-channel architectures; (ii) operation simplicity, eliminating the need for viscous fluid pretreatment or sheath flow assistance; (iii) intrinsic scalability achieving milliliter-per-minute throughput without parallelization; and (iv) exceptional focusing stability maintaining consistent performance during prolonged operation. This innovation has garnered notable recognition (Fig. 4C) (*74*, *89*, *95*, *96*), including a highlight feature by *Advanced Science News* titled “Novel microfluidics strategy for efficient fluid manipulation.” Subsequent adaptations by independent groups have expanded its utility to CTC isolation (*97*, *98*), single-cell analysis (*99*–*101*), cell washing (*102*), and exosome purification (*103*). These advancements stem from the platform’s inherent architectural compactness, rational spatial utilization, operational robustness, and throughput insensitivity, collectively positioning it as a transformative solution for high-throughput cellular sorting workflows.

Inertial microfluidics offers distinct advantages over conventional microfluidic approaches: Its separation dynamics governed solely by flow rate modulation simplifies operational workflows, a simple channel reduces fabrication complexity and cost while enabling multiphysical field integration, and intrinsic high-throughput processing enhances sample handling efficiency without compromising biocompatibility. However, two inherent limitations persist: inadequate resolution for cellular subpopulations with minimal size differences and restricted applicability to mechanically homogeneous samples. Emerging strategies focus on hybrid parameter-coupled sorting (e.g., active-inertial cascading and DLD-inertial hybrid architectures) to enhance resolution thresholds, complemented by computational fluid dynamics–guided optimization of flow-curvature matching paradigms to transcend current performance boundaries.

### Viscoelastic microfluidics

**Fig. 5.**
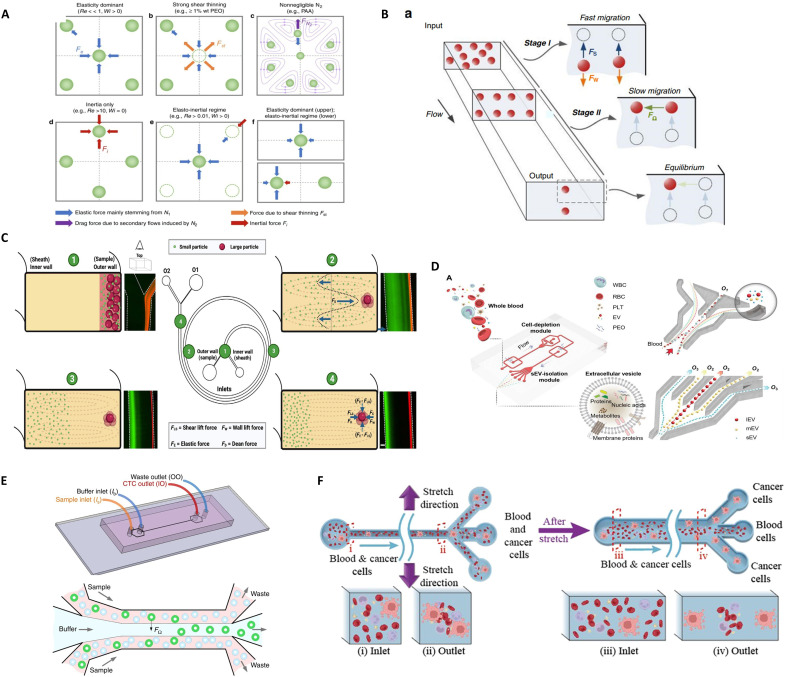
Viscoelastic microfluidics. (**A**) Reynolds number–dependent focusing behavior of microspheres in inertial microfluidics. Image credits: Reused with permission from (*104*) (CC BY 4.0; https://creativecommons.org/licenses/by/4.0/deed.en). (**B**) Hydrodynamic forces on microspheres in viscoelastic microfluidic flows. Image credits: Reused with permission from (*107*). Used with permission of Royal Society of Chemistry from (*107*); permission conveyed through Copyright Clearance Center Inc. (**C**) Spiral inertial and viscoelastic microfluidics for direct bacterial sorting from whole blood. Image credits: Reused with permission from (*111*) (CC BY-NC 4.0; https://creativecommons.org/licenses/by-nc/4.0/). (**D**) A label-free isolation strategy for sEVs is realized using a viscoelasticity-based microfluidic chip, bypassing the need for biomarkers or external fields. Image credits: Reused with permission from (*109*) (CC BY 4.0; https://creativecommons.org/licenses/by/4.0/deed.en). (**E**) Schematic of CTC enrichment from blood via viscoelastic focusing, where CTCs are selectively aligned and separated from hematopoietic cells. Image credits: Reused with permission from (*108*) (CC BY 4.0; https://creativecommons.org/licenses/by/4.0/deed.en). (**F**) Ultrastretchable viscoelastic microfluidics enable deformability-based tumor cell sorting, extending applicability to rare cell isolation under extreme flow conditions. Image credits: Reused with permission from (*112*). Reprinted in part with permission from (*112*). Copyright 2024 American Chemical Society.

Viscoelastic microfluidics exploits the unique rheological properties of non-Newtonian fluids containing polymeric additives to achieve precise particle manipulation (Fig. 5A) (*104*). These fluids exhibit dual viscous and elastic characteristics, enabling size- and deformability-dependent particle migration through spatially varying stress fields (*105*). In straight microchannels under low Reynolds number regimes, elastic stress dominance drives particles toward low-shear regions (e.g., channel centerline) via normal stress difference–induced focusing (Fig. 5B) (*106*, *107*). At elevated flow rates where inertial effects become nonnegligible, synergistic interactions between elastic lift forces and secondary Dean vortices in curved geometries enhance 3D focusing precision. Crucially, channel architecture dictates equilibrium positions: Rectangular cross sections produce narrowed focusing bands with increasing aspect ratios, while curvature-induced secondary flows modulate migration trajectories (*105*). The technology’s ability to decouple flow control from external fields, combined with shear thinning–mediated viscosity modulation, establishes it as a robust platform for label-free bioparticle separation with inherent scalability (Fig. 5E) (*108*).

Mounting experimental evidence positions small extracellular vesicles (sEVs) as crucial mediators in disease pathogenesis and therapeutic development, particularly for cancer progression, infectious processes, and neurodegenerative disorders. A breakthrough viscoelastic microfluidic chip engineered by Meng *et al.* (*109*) features two cascaded separation modules—a cellular depletion unit followed by an exosome isolation unit—enabling label-free isolation of sEVs directly from whole blood (Fig. 5D). This platform achieves exceptional performance metrics, with isolation purity exceeding 97% and recovery rates surpassing 87%, notably advancing current clinical sample processing capabilities. This technological advancement addresses long-standing limitations in extracellular vesicle research by providing standardized isolation from complex biofluids. Wang *et al.* (*110*) developed a label-free cell separation platform using microchannels with oblique ridges, leveraging differential viscoelastic responses during repeated compression-relaxation cycles. This design induces shape recovery dynamics where weakly viscoelastic cells rapidly regain morphology and undergo lateral displacement along the ridges, while strongly viscoelastic cells exhibit delayed recovery and remain confined by hydrodynamic recirculation. Concurrently, Narayana Iyengar *et al.* (*111*) pioneered a viscoelastic-inertial microfluidic system for high-resolution bacterial isolation from whole blood, achieving unprecedented throughput (1 ml/min) for downstream phenotypic and molecular analyses (Fig. 5C). Their spiral channel architecture exploits size-dependent particle migration: Larger blood cells migrate toward the outer wall through inertial focusing, while smaller bacteria (<3 μm) are selectively entrained in Dean vortices. The system demonstrates exceptional separation efficiencies of 96% for 1-μm particles and complete recovery (100%) for 3-μm targets. In clinically relevant models using *Escherichia coli*–spiked blood samples, continuous separation efficiencies of 82 to 90% were maintained across various hemodilution levels, with processing times scaling from 40 min (82% efficiency) to 3 hours (90% efficiency) per milliliter of sample. This label-free approach eliminates biochemical modification requirements while maintaining operational simplicity and cost-effectiveness. In addition, to overcome the bottleneck of traditional rigid microfluidic devices that require repeated processing and testing when optimizing key channel geometrical parameters such as size and aspect ratio, Kang *et al.* (*112*) use hyperelastic, biocompatible material to create flexible microfluidic devices that dynamically tune microchannel dimensions in real time via external stretching, notably streamlining device optimization for specific separation targets like particles or cells, demonstrated successfully for particle migration control and separation in viscoelastic flows within channels with an aspect ratio of 3 (Fig. 5F).

Viscoelastic microfluidics has emerged as a transformative cell sorting platform, leveraging its high-resolution discrimination, adaptable channel geometries, and multimodal sorting capabilities driven by synergistic size-stiffness modulation. However, scalable adoption faces three critical barriers: (i) labor-intensive viscoelastic medium preparation requiring precise polymer formulation control, (ii) resolution limitations for cellular subpopulations with near-identical biophysical properties, and (iii) suboptimal throughput-cost ratios compared to inertial microfluidic counterparts. Emerging strategies aim to overcome these constraints through in situ polymerization protocols for standardized medium synthesis, hybrid architectures integrating dielectrophoretic tuning, and optorheological systems enabling real-time viscoelasticity modulation. These innovations collectively address the precision-scalability trade-off, advancing its translation toward clinical diagnostics and industrial-scale bioprocessing.

## SHORTCOMINGS OF MICROFLUIDIC CELL SORTING

In summary, microfluidic cell sorting technology has demonstrated tremendous application potential in rare cell separation, single-cell analysis, and clinical diagnosis owing to its high throughput, low sample consumption, and excellent biocompatibility (*15*, *113*). However, its practical application still faces multiple challenges. Traditional microfluidics–based cell sorting primarily relies on manually designed feature extraction, in which experts select morphological indicators or signal characteristics of cells on the basis of prior experience. This process is not only time-consuming and labor-intensive but also prone to human bias and error. For example, CTCs and WBCs of comparable size are difficult to distinguish, and deformability assessment based on migration trajectories is highly dependent on specific chip structures and flow rates; once these conditions vary, the generalizability is markedly reduced. Although some approaches incorporate basic image processing techniques to assist identification, the application of ML remains relatively superficial, failing to fully exploit the deeper features and high-level semantic information of cells. A particularly critical issue lies in the high heterogeneity of clinical samples. Cellular debris, protein aggregates, and other impurities not only cause microchannel blockage or flow-field disturbances but also amplify noise, substantially compromising the separation purity of principles that rely on stable fluid dynamics—such as inertial focusing and DLD. Current microfluidic systems lack the ability to intelligently detect and adapt to such abnormalities. Furthermore, handcrafted features exhibit poor robustness and are easily affected by experimental variability and sample impurities. Traditional image processing methods rely heavily on manually set parameters and are unable to overcome measurement errors caused by uneven illumination, defocus blur, and debris occlusion; flow rate fluctuations or nonspecific adsorption of biological molecules will further interfere with the extraction and analysis of cell movement trajectories, making features that rely on fixed thresholds or absolute displacement invalid, notably limiting the accuracy and repeatability of the system.

In recent years, diverse computational approaches have been rapidly adopted to tackle these challenges. Traditional ML algorithms accurately classify cell types using morphological, mechanical, or spectral features, increasing automation and objectivity in cell sorting. DL models—including convolutional neural networks (CNNs), You Only Look Once (YOLO)–based architectures, and residual networks (ResNets)—extract local texture and morphological characteristics from cell images, boosting classification accuracy among similar-sized cells and demonstrating robust performance and adaptability in complex biological contexts. These innovations offer a compelling technical foundation for label-free cell sorting. In addition, Bayesian optimization has been used to refine microfluidic chip design, further enhancing sorting efficiency.

Therefore, the microfluidic sorting system integrating computing strategies is expected to achieve adaptive optimization of sorting parameters, real-time discrimination, and decision support, thereby improving the system robustness and cross-scenario applicability. Further integration with computationally driven chip design methods will drive the evolution of microfluidic technology toward customization, high performance, and integration, providing key technical support for its application in precision medicine and clinical transformation.

## ML-DRIVEN CELL SORTING

The exponential growth in cellular datasets generated by microfluidic systems has necessitated advanced analytical frameworks to decode complex phenotypic signatures. ML addresses this challenge by learning to extract latent patterns from training data to enable accurate classification or predictive modeling of cellular behaviors, thereby enhancing sorting precision and diagnostic utility in clinical workflows (*114*, *115*).

Traditional ML approaches [e.g., support vector machines (SVMs) and *K*-nearest neighbors (KNNs)] rely on manually designed features for cell characterization, yet their limited generalizability often fails to capture intricate cellular heterogeneity (*116*). DL architectures circumvent this bottleneck through autonomous, multilayered feature extraction, enabling comprehensive representation of multiscale morphological and textural patterns critical for distinguishing rare cell subtypes (*117*, *118*). This section systematically examines ML-empowered microfluidic sorting through two complementary algorithm systems: traditional ML and DL, with emphasis on their mechanistic implementations and translational validations on biomedical applications.

### Traditional ML–based cell sorting

**Fig. 6.**
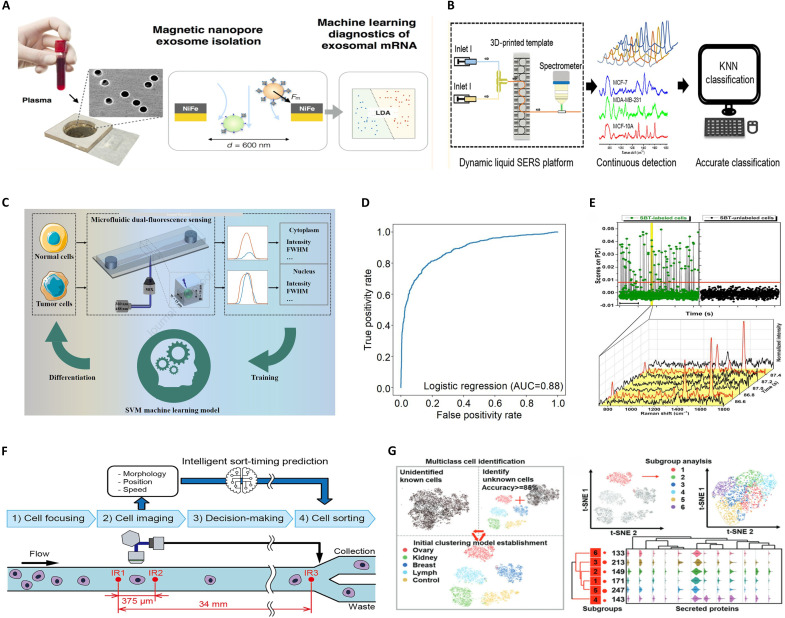
Traditional ML algorithms have been extensively integrated with microfluidic systems to enhance diagnostic and analytical capabilities. (**A**) LDA combined with microfluidic technology achieves high-specificity exosome profiling for pancreatic cancer diagnosis. Image credits: Reprinted in part with permission from (*126*). Copyright 2017 American Chemical Society. (**B**) The KNN algorithm is used for real-time pattern recognition in microfluidic imaging data, optimizing cell sorting accuracy. Image credits: Reprinted in part with permission from (*129*). Copyright 2019 American Chemical Society. (**C**) SVM-assisted dual-fluorescence FCM enables bladder tumor cell detection on the basis of morphological features within microfluidic platforms. Image credits: Reused with permission from (*137*). Reprinted from (*137*); copyright (2024) with permission from Elsevier. (**D**) Receiver operating characteristic (ROC) curves and area under the curve (AUC) metrics validate the performance of LR models in classifying cellular phenotypes under dynamic flow conditions. Image credits: Reprinted in part with permission from (*133*). Copyright 2023 American Chemical Society. (**E**) Distinguishing cell signals with SERS tags from background noise signals via the PC1 scores derived from generalized PCA. Image credits: Reprinted in part with permission from (*159*). Copyright 2015 American Chemical Society. (**F**) Ensemble learning frameworks, including XGBoost models, enable intelligent classification and time-resolved prediction of pancreatic cancer biomarkers using exosome-derived data from nanofluidic isolation systems. Image credits: Reused with permission from (*145*). 2022 International Society for Advancement of Cytometry. (**G**) Tumor cell clustering and subgroup analysis are automated through ML-driven image processing, facilitating phenotypic heterogeneity studies in cancer populations. Image credits: Reused with permission from (*152*). Copyright 2022 Wiley-VCH GmbH.

Traditional ML is capable of learning regularities from data and subsequently making predictions, classifications, or decisions. These algorithms are systematically divided into supervised and unsupervised learning on the basis of their training methodologies (Fig. 6) (*119*). Supervised approaches, such as SVM and KNN, use manually annotated training data and have been proven instrumental in microfluidic-assisted cell sorting. Unsupervised learning, in contrast, leverages unlabeled data to train models that uncover hidden patterns and structures within the data, thereby enabling cell sorting through self-organized feature discovery (Table 2) (*120*).

**Table 2.** Microfluidic cell sorting based on traditional machine learning.

Category	Technique	Microfluidic processing	Sample to separate	Accuracy	Throughput	References
Supervised learning	LDA	Active	MCF-7 cells	60%	6 cells/min	(*124*)
	LDA	Passive	Tumor cells (T24, U251, Panc02, WBCs)	>90%	16.20 ± 0.06 min	(*125*)
	LDA	Active	Exosomal RNA data	–	10 ml/hour	(*126*)
	KNN	Active	MCF-10A, MCF-7, MDA-MB-231 cells	94.1 ± 1.14%	0.004 m/s	(*129*)
	KNN	Active	Yeast cells (unbudded, small-budded, large-budded)	[86.2%, 99.5%]	12 cells/min	(*130*)
	Regression model	Passive	MDA-MB-231 and MCF-10A cells	93%	500 cells/s	(*132*)
	Logistic regression	Passive	MCF-7 cells	99.4%	135 μl/min	(*133*)
	Logistic regression	Passive	CAFs, A549, and H322 cells	66.7%	High throughput	(*134*)
	SVM	Passive	Bladder cancer cells	84.7%	High throughput	(*137*)
	SVM	Passive	Ovarian cancer cells (A2780)	92.84%	Low throughput	(*138*)
	SVM	Passive	CD4+ and CD8+ T lymphocytes	79% (unstimulated), 88% (stimulated)	–	(*139*)
	SVM	Passive	MCF-10A and MDA-MB-231 cells	–	280 cells/s	(*140*)
	SVM	–	WBCs, SW480, DLD-1, HCT116, Panc-1, HepG2	96.8%	Low throughput	(*141*)
	EBT	Passive	Circulating tumor cell clusters (CTCCs)	95.35%	3 μl/min	(*144*)
	XGBoost	Active	MCF-10A and MDA-MB-231 cells	–	2000 eps	(*145*)
	Random forest	Passive	Ovarian cancer	55.4%	–	(*146*)
	Random forest	Passive	CD34+ HSCs	79.6%	1000 cells/s	(*147*)
Unsupervised learning	*K*-means	Passive	Secreted biomarkers of tumor cells	95.0%	High throughput	(*152*)
	*K*-means	Passive	Plasma samples	–	5 μl/min	(*153*)
	PCA	Active	Colonic biopsy samples (healthy vs tumor)	–	100–1000 cells/s	(*157*)
	PCA	Passive	Cancer cells	86.99% (1D), 88.61% (2D)	100–300 μl/hour	(*158*)
	PCA	Passive	Prostate cancer cells	–	7–8 cells/s	(*159*)

### Supervised learning–enabled cell sorting

By analyzing the intrinsic relationship between input features and output labels, supervised learning constructs predictive or classificatory models through labeled training datasets (*121*). The training process of supervised learning involves data preprocessing, model selection, training implementation, performance evaluation, optimization, and fine-tuning in ML (*122*). This paradigm has demonstrably enhanced the accuracy and throughput of cancer-normal cell sorting in microfluidic systems while exhibiting robust scalability in multicellular recognition tasks.

### Linear discriminant analysis

Linear discriminant analysis (LDA), also known as Fisher’s linear discriminant, is a foundational supervised learning algorithm in pattern recognition and ML. Its core principle lies in identifying an optimal projection subspace that maximizes interclass difference while minimizing intraclass difference within dimensionally reduced data representations (*123*). This discriminative classification framework has been successfully implemented in processing Raman spectroscopy, surface-enhanced Raman scattering (SERS) signals, and quantitative polymerase chain reaction datasets for cellular sorting applications, which leverages intrinsic spectral signatures and gene expression profiles of cells or exosomes.

Dochow *et al.* (*124*) developed dual-platform microfluidic systems (quartz capillaries and glass chips) for Raman spectral acquisition of a single cell, achieving overall classification accuracies of 92.2% for erythrocytes, leukocytes, BT-20, OCI-AML3, and MCF-7 cells in capillary systems and 94.9% for leukocytes, BT-20, MCF-7, and OCI-AML3 cells in chip; both systems use the LDA algorithm for classification following the spectral processing. Xie *et al.* (*125*) used spiral inertial microfluidic chips with a sorting purity exceeding 80% in combination with LDA-classified SERS to sort bladder cancer (T24), glioma (U251), murine pancreatic cancer (Panc02) cells, and leukocytes (WBCs). The results demonstrated that the constructed classification model achieved more than 90% discrimination accuracy, indicating high confidence and reliability, thereby notably enhancing the efficiency of cell sorting. Ko *et al.* (*126*) proposed a method based on the microfluidic technology and LDA algorithm for pancreatic cancer diagnosis; the LDA algorithm is primarily used to extract classification information from exosomal RNA data, generating predictive models capable of distinguishing between healthy, precancerous, and cancerous conditions, thereby improving diagnostic accuracy and specificity (Fig. 6A).

LDA is an efficient linear classification and dimensionality reduction method, and when the cell data are linearly separable and the distribution approximates a normal distribution, LDA offers the advantage of high computational efficiency, particularly excelling in scenarios with clear classification boundaries and small sample cell data. However, LDA has strict data distribution assumption and limited dimensionality reduction capabilities and is sensitive to nonlinear data and noise. In applications that involve handling nonlinear cell features or require good robustness, algorithms such as KNN can demonstrate their advantages in adapting to complex data distributions.

### *K*-Nearest neighbor

The KNN algorithm is a classical instance-based learning method and is extensively applied in classification and regression tasks (*127*). On the basis of instance-based learning, the distance between the sample to be classified and the samples in the training set is computed, and then the majority voting (classification) or local averaging (regression) is conducted among the *k*-closest neighbors (*128*).

Notably, KNN excels in cellular classification by leveraging intrinsic biophysical properties encoded in spectral signatures (e.g., SERS spectra) or imaging features (e.g., fluorescence patterns). Xu *et al.* (*129*) implemented a flexible tube–based microfluidic platform with dynamic liquid-phase SERS and then achieved a 94.1 ± 1.14% classification accuracy for normal breast epithelial cells (MCF-10A) versus breast cancer subtypes (MCF-7 and MDA-MB-231) via KNN-driven spectral analysis (Fig. 6B). Yu *et al.* (*130*) developed an integrated, microvalve-assisted microfluidic platform that extracts morphometric features from individual yeast cells—specifically, the presence or absence of budding and the relative bud size—and uses a KNN classifier to assign cells to unbudded, small-budded, or large-budded subpopulations. The system operates at a throughput of 12 cells per minute, achieving a sorting probability with a confidence interval of 86.2 to 99.5%.

KNN is a nonparametric method that has the advantages of not requiring assumptions about the cell data distribution. It performs well in scenarios where class domains intersect or overlap. However, it has disadvantages such as high computational complexity, large storage requirements, sensitivity to the selection of *K* value, susceptibility to noise and outliers, and lack of model interpretability. In terms of computational efficiency, model interpretability, and noise robustness, algorithms such as logistic regression (LR) optimize parameters by maximizing the likelihood function, thereby enhancing robustness to noise and outliers and effectively compensating for the shortcomings of KNN.

### Logistic regression

LR is a generalized linear model and operates by transforming linear predictor outputs through sigmoidal nonlinearity (e.g., logistic function), mapping continuous values into probabilistic estimates within the [0, 1] interval. This framework models conditional probabilities for binary classification via maximum likelihood estimation, where parameter optimization maximizes the log-likelihood function of observed data to predict event occurrence probabilities (*131*). In cellular sorting applications, LR classifiers leverage biophysical signatures—including cellular cross-sectional area, deformability metrics, and dielectric polarization characteristics—to establish decision boundaries, which would discriminate cell populations on the basis of their intrinsic phenotypes or mechanical characteristics.

Chen *et al.* (*132*) developed an inertial multiforce deformability cytometry platform integrated with LR classifiers, achieving high-throughput cellular deformability profiling at nearly 500 cells per second with a classification accuracy of 93%. However, when using this algorithm to sort MCF-7 and MCF-10A cells, the average sorting efficiency was around 70%. Chen *et al.* (*133*) engineered a viscoelastic sorting–integrated deformability cytometer for label-free tumor cell selection and mechanical phenotyping, demonstrating more than 95% sorting efficiency and 81% purity (Fig. 6D). Their LR-based multiparametric analysis (cell area and deformability metrics) distinguished tumor cells from leukocytes with 97% discrimination accuracy, reaching the specificity of 99.4% for MCF-7 identification. Wang *et al.* (*134*) proposed the microfluidics-based polarization microscopy imaging analysis method, achieving 66.7% classification accuracy between cancer-associated fibroblasts (CAFs) and non–small cell lung cancer cells (A549 and H322).

LR could be used on binary and multiple class classification tasks, given that it exhibits inherent advantages including computational efficiency, structural simplicity, noise robustness, and intuitive interpretability. By leveraging its fast-processing capabilities for large-scale cell datasets and mitigating overfitting through regularization, the model performs well in the rare cell populations such as CTCs. In contrast, LR has a limited capacity for modeling nonlinear decision boundaries, and it relies heavily on feature engineering. It is also susceptible to the “curse of dimensionality” in high-dimensional data. In terms of processing the generalization of high-dimensional data, SVM could effectively capture nonlinear patterns by using a kernel function to project the data into a higher-dimensional space and maximizing the classification interval and thus exhibit its ability on generalization performance.

### Support vector machines

The method of SVM is widely used for classification and regression analysis (*135*). The principle aims to identify an optimal hyperplane that maximizes the distance between sample points of different classes and the decision boundary (*136*). In microfluidic cell sorting applications, SVM classifiers leverage multidimensional phenotypic signatures (e.g., morphometric, biomechanical, or spectral profiles) and hyperplane-projected feature space partitioning to achieve label-free isolation of target subpopulations with high efficiency.

Zhang *et al.* (*137*) implemented a dual-fluorescence FCM platform, and with SVM optimization, a peak classification accuracy of 84.7% is achieved on bladder cancer cell classification in the urine specimens (Fig. 6C). Su *et al.* (*138*) established an analytical framework combining histogram of oriented gradients–based feature extraction and SVM classifiers to decode 2D light-scattering patterns of ovarian cancer cells (A2780) and normal ovarian epithelial cells (HOSEpiC), demonstrating a 92.84% discrimination accuracy. Rossi *et al.* (*139*) developed a microfluidic photonic scattering system using cubic SVM to analyze the biophysical signatures of CD4+/CD8+ T lymphocytes, achieving a baseline classification accuracy of 79%, which improved to 88% upon selective stimulation with antiapoptotic proteins. However, the ML accuracy using a red laser was 68.49%, while the accuracy with a blue laser was 79.01%. Liang *et al.* (*140*) engineered a label-free phenotyping system integrating hydrodynamic cell stretching. With the help of SVM classifiers, they achieved comparable performance to fluorescence-activated sorting, including SVM-predicted MCF-10A: MDA-MB-231 ratios of 0.9:1 (versus FCM-measured 0.97:1) and 5.03:1 (versus FCM-measured 5.33:1) in 1:1 and 5:1 mixed populations, respectively. Ozaki *et al.* (*141*) leveraged histogram of oriented gradients–derived subcellular texture features and SVM to discriminate human WBCs from five carcinoma lines (SW480, DLD-1, HCT116, Panc-1, and HepG2), attaining a 96.8% identification accuracy.

For its powerful high-dimensional data processing ability, the flexibility of the kernel function, model simplicity, and flexible parameter tuning, SVM has shown outstanding performance in classifying feature data of rare cell types, including CTCs and complex cells. Despite its advantages, SVM struggles with high computational complexity and notable memory consumption when dealing with large amounts of cell sample data derived from a high-throughput system. In addition, SVM is sensitive to the choice of kernel function and parameters, making it difficult to directly support multiclass tasks, and it is also more sensitive to noise and outliers. Now, ensemble learning methods (such as random forest and XGBoost) have demonstrated potential in adapting to complex, large-scale, and multiclass task scenarios.

### Ensemble learning

According to combining several weak learners to form a strong learner, ensemble learning enhances the overall performance (*142*), such as improving prediction accuracy and reducing the risk of overfitting (*143*). Vora *et al.* (*144*) conducted a study on the detection of circulating tumor cell clusters (CTCCs) based on confocal backscatter and fluorescence FCM. They used the ensemble boosted tree (EBT) model for the analysis and detection of CTCCs and achieved an average sensitivity (standard deviation) of 92.51 ± 2.29%, a specificity of 95.94 ± 0.69%, a purity of 82.78 ± 2.05%, and an accuracy of 95.35 ± 0.28%. Zhao *et al.* (*145*) proposed an intelligent sorting timing prediction strategy based on the XGBoost algorithm. By inputting cellular morphological features into the XGBoost model, the prediction error of sorting time was reduced by 41.5% compared to the previous flow rate–based method, thereby almost doubling the sorting event rate on intelligent image-activated cell sorting (Fig. 6F). Li *et al.* (*146*) proposed a nano-FCM method based on aptamers for molecular detection and classification of ovarian cancer by analyzing tumor markers on sEVs. In this study, the random forest algorithm was applied in conjunction with the expression data of multiple protein markers, and an overall classification accuracy of 55.4% in the validation cohort was achieved. Herbig *et al.* (*147*) used the random forest–assisted real-time deformability cytometry technology to analyze the morphological and mechanical properties of CD34+ hematopoietic stem cells (HSCs) in bone marrow, successfully distinguishing healthy samples from those diagnosed with myelodysplastic syndrome. The out-of-bag accuracy was 82.9% along with the average validation accuracy of 79.6%.

Ensemble learning reduces the risk of overfitting and enhances generalization capability by combining multiple base learners. Ensemble learning helps to capture different patterns in complex data and support parallel processing, thereby enhancing computational efficiency. Ensemble learning enhances robustness by reducing the reliance on the parameters of a single model, while in contexts requiring efficient computation, improved generalization, and reduced overfitting, DL could leverage deep neural networks to automatically learn features without relying on multimodel combinations.

In summary, comparative analysis across critical performance metrics—classification capacity, computational efficiency, generalization capability, and nonlinear data adaptability—reveals that LDA remains constrained by linear separability assumptions and limited processing capability on high-dimensional data, so it is hard to resolve complex cellular phenotypic distributions. KNN faces issues of computational complexity and sensitivity to noise, and it shows suboptimal performance under conditions of class imbalance. LR, while computationally frugal and interpretable, exhibits underfitting tendencies in nonlinear classification landscapes. SVM excels in high-dimensional feature space navigation with strong generalization bounds but demands long training times and is sensitive to parameters. On the basis of the synergistic architecture of learner integration, ensemble learning achieves accuracy-stability equilibrium and nonlinear pattern adaptability, and it could be treated as a versatile paradigm on multiplexed cellular classification tasks.

### Unsupervised learning–guided cell sorting

By using techniques such as density estimation, clustering, and dimensionality reduction, unsupervised learning operates on label-free datasets to autonomously uncover latent data structures through statistical feature analysis and similarity metrics (*148*). Its central objective is to derive intrinsic cellular patterns and organizational representations without label-dependent supervision. In cellular analytics, this paradigm enables the uncovering of meaningful patterns and biophysical structures from the unlabeled tumor cell, thereby showing more interpretation and gaining deeper insights into the complexity and heterogeneity of cells (*149*, *150*).

### *K*-Means clustering

The principle of the *K*-means algorithm, a cornerstone clustering methodology, lies in dividing the dataset into *K* clusters and achieving this by minimizing the sum of the distances from each data point to the center of its respective cluster (*151*). The *K*-means algorithm is notable for computational simplicity, scalability, and interpretable cluster topology, and it effectively conducts clustering analysis by using the spatial and feature information of the complex datasets like cellular secretomic profiles and optical coherence tomography features.

Wang *et al.* (*152*) developed a high-throughput live-cell secretomic profiling platform integrating microfluidic chips with *K*-means clustering, and through the multivariate analysis of single-cell secretory signatures, they achieved 95.0% classification accuracy for tumor cell subtypes across thousands of cellular events (Fig. 6G). Chen *et al.* (*153*) engineered a deformable nanoscale sieve system based on microbead stacking and used the *K*-means algorithm to process optical coherence tomography datasets, and then they achieved the precise segmentation of nanoscale sieve architectures and their volumetric and topographic quantification.

The *K*-means algorithm is simple, easy to implement, and computationally efficient, which makes it suitable for large-scale datasets. Also, its clustering results are interpretable, so it offers a clear visualization of data distribution. However, the *K*-means algorithm is sensitive to the initial selection of centroids, which can lead to local optima, and requires specifying the number of clusters, *K*, in advance. To address the limitation of the lack of clear criteria for selecting the value of *K*, methods such as DBSCAN can be used to enhance the stability, robustness, and applicability (*154*).

### Principal components analysis

Principal components analysis (PCA) is a widely used data analysis and dimensionality reduction technique across multiple fields (*155*). On the basis of linear transformations, it projects high-dimensional data onto principal components ordered by variance, effectively reducing dimensionality to simplify the data structure, which is applied in various fields, such as computer vision and bioinformatics. PCA is useful for dimensionality reduction, and especially in cellular analytics, PCA enables efficient distillation of multidimensional mechanophenotypic signatures—including cellular biomechanical phenotypes (elastic modulus and deformability), hyperspectral imaging features, and Raman spectral fingerprints—into principal component subspaces, thereby facilitating robust discrimination of cellular subtypes and functional states in label-free sorting workflows (*156*).

Soteriou *et al.* (*157*) used microfluidic technology for rapid diagnosis based on real-time fluorescence and deformability cytometry. In this study, the PCA algorithm was used for dimensionality reduction of physical phenotype data from mouse and human colon biopsy samples, and then they effectively distinguished healthy tissue from tumor tissue. Jagannadh *et al.* (*158*) developed a label-free cell sorting and recognition method based on microfluidic microscopy. They used the PCA algorithm to extract cellular morphological features and generate cell-specific signatures, combining them with an SVM classifier; they achieved the classification accuracy of 86.99% for 1D PCA and 88.61% for 2D PCA. Pallaoro *et al.* (*159*) designed a detection platform combining microfluidics and SERS. First, gating PCA was used for data preprocessing to filter out cell-related spectral data. Then, PCA was applied to the Raman spectroscopy data to distinguish cancer cells from normal cells (Fig. 6E).

The PCA algorithm can map high-dimensional data to a lower-dimensional space through linear transformations without data labels. However, the classic PCA algorithm has limited capability in handling nonlinear data and noise in cell data, has poor interpretability of the reduced features, and faces difficulties in determining the optimal number of principal components. By combining methods such as kernel PCA, robust PCA, and feature selection with PCA, the PCA algorithms could handle nonlinear data, missing or outlier values, complex manifold structures, and high-dimensional redundant features, thereby improving the dimensionality reduction performance (*160*, *161*).

It is evident from the previous analysis that traditional ML can automatically process the vast amounts of data generated during microfluidic operations, thereby enabling the system to make independent sorting decisions on the basis of recognition outcomes. Furthermore, ML enhances sorting efficiency; these models can rapidly analyze high-dimensional data such as microscopic images, uncovering patterns imperceptible to the human eye and thus accelerating sorting while preserving accuracy.

Microfluidic cell sorting systems enhanced by traditional ML still face numerous limitations and challenges. These systems rely heavily on large volumes of high-quality training data; however, subjective variability in feature extraction and annotation across experimenters makes it difficult to capture the full spectrum of cellular phenotypes. As a result, models are often constrained to specific data distributions, leading to performance degradation in broader applications. Data imbalance further compounds this issue: When rare cell types are underrepresented in the training set, models tend to favor the majority class, reducing their ability to identify rare cells. In addition, significant variations in microfluidic devices and operating conditions across laboratories result in heterogeneous data distributions. Although many models perform well in single-laboratory settings, their cross-platform generalization remains insufficient, limiting the reproducibility and scalability of these approaches. This issue is exacerbated by the reliance on self-constructed datasets, where models often show markedly reduced performance on cell types with limited sample sizes. Last, the integration of algorithms with hardware poses a critical challenge. Microfluidic sorting requires millisecond-level decision-making, imposing strict constraints on computational latency, yet embedding complex DL models into microfluidic chips—particularly portable edge devices—remains technically demanding.

## DL-POWERED CELL SORTING

**Fig. 7.**
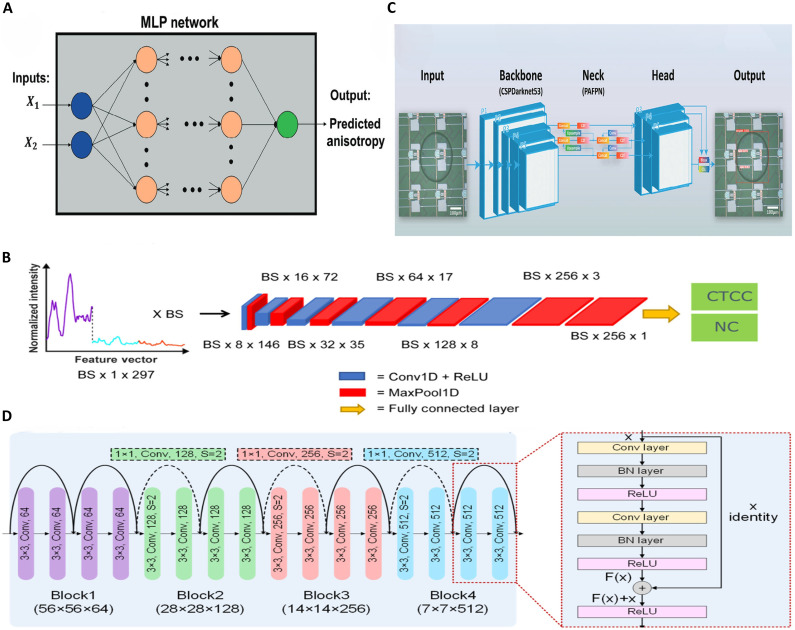
DL architectures. (**A**) MLP. Image credits: Reused with permission from (*171*) (CC BY-NC 4.0; https://creativecommons.org/licenses/by-nc/4.0/). (**B**) Using a 1D feature vector of normalized scattering intensities from three distinct wavelengths, coupled with a CNN, enables the distinction of CTCC peaks from non-CTCC peaks. Image credits: Reused with permission from (*175*) (CC BY-NC 3.0; https://creativecommons.org/licenses/by-nc/3.0/). (**C**) YOLOv8 is used to sort HeLa cells from red blood cells, isolate cancer cells from WBCs, and distinguish WBC subtypes. Image credits: Reused with permission from (*182*) (CC BY 4.0; https://creativecommons.org/licenses/by/4.0/). (**D**) Residual blocks are designed to facilitate complex feature learning and mitigate gradient degradation in deep networks. Image credits: Reused with permission from (*186*). Reprinted from (*186*); copyright (2024) with permission from Elsevier.

DL is an ML methodology that uses hierarchical architectures for feature extraction and pattern recognition. Through hierarchical abstraction and automated learning mechanisms, DL can derive sophisticated data representations directly from original inputs, thereby enabling automated processing and decision-making in complex tasks (Fig. 7) (*162*). Neural network models in DL simulate interconnected processing units of biological nervous systems. These artificial networks perform intricate nonlinear transformations of input data through coordinated interlayer computations, achieving good results in classification, regression, pattern recognition, and other machine intelligence tasks (*163*). Multilayer perceptron (MLP) constitutes the foundational neural architecture using fully connected layers to establish nonlinear mappings, which makes it suitable for elementary pattern recognition challenges. CNNs leverage convolutional and pooling operations to capture localized spatial features, demonstrating high efficacy in image analysis and computer vision applications (*164*). Deep neural networks encompass architectures with multiple hidden layers capable of modeling intricate nonlinear relationships (*165*). YOLO frameworks represent a series of CNN-based object detection systems optimized for real-time multiobject identification in visual data streams (*166*). Notably, ResNets address gradient degradation in deep architectures through skip-connection modules, substantially enhancing model performance in deep-layer configurations (*167*). These advanced DL architectures have demonstrated effectiveness in complex pattern recognition and classification tasks, particularly in microfluidic cell sorting applications (Table 3).

**Table 3.** Microfluidic cell sorting based on deep learning.

Category	Technique	Microfluidic processing	Sample to separate	Hidden layers	Accuracy	Throughput	Reference
Deep neural networks	MLP	Passive	Live pancreatic cancer cells and CAFs	30	–	6 μl/min	(*171*)
	CNN	Passive	Circulating tumor cell clusters (CTCCs)	6 convolutional blocks with max-pooling	72%	3 μl/min	(*175*)
	CNN	Passive	Tumor cells (holographic microscopy)	8	–	3.5 ml/min	(*176*)
	CNN	Passive	Pancreatic ductal adenocarcinoma (PDAC)	6	97%	–	(*177*)
	CNN	Passive	RBCs and WBCs	6	98.57%	–	(*178*)
	CNN/MLP	Active	Mouse retinal cells	–	97.4%	1000 cells/s	(*179*)
	CNN	Active	HeLa cells	YOLOv8	98.5%	5 cells/s	(*182*)
	CNN	Passive	RBCs	YOLOv4	89.24%	10 μl/min	(*183*)
	CNN	Passive	Cancer cell lines (HT29, A549, KYSE30)	YOLOv4	99.40%, 99.52%, 99.47% (AP)	50,000 cells/min	(*184*)
	CNN	Passive	PC3 prostate cancer cells	YOLOv5	–	–	(*185*)
	ResNet	Passive	PBMCs, RBCs, and cancer cells	–	91.7%	–	(*187*)
	ResNet	Passive	Lung cancer cells	–	99.77%	–	(*188*)
	ResNet	Active	HL-60, Jurkat, and K562 cells	18	95.1 & 94.2% purity	82.8 eps	(*115*)

### Multilayer perceptron

MLP is a classical artificial neural network architecture (*168*), and it integrates an input layer, several hidden layers, and an output layer through weighted interconnections. Its operational processing involves forward propagation generating predictions, sequential nonlinear transformations (enabled by activation functions such as Sigmoid or ReLU), and backward propagation operation, which aims to adjust synaptic parameters with the gradient descent method (*169*). MLP’s structural simplicity and versatility make it well suited for regression and classification tasks without requiring complex preprocessing.

Talebjedi *et al.* (*170*) integrated MLP with a multiobjective heuristic optimization approach to propose a previously unidentified automated design methodology for acoustofluidic devices. They fabricated an acoustic microfluidic chip and established an experimental setup using an MLP model that takes IDT geometric features as inputs and outputs reflection coefficient and quality factor. Posttraining evaluation demonstrated the model’s exceptional performance, achieving *R*^2^ values of 0.97 for the reflection coefficient prediction and 0.96 for the quality factor estimation. Jarmoshti *et al.* (*171*) used an MLP neural network to process original impedance signals that exhibit diverse shapes (Fig. 7A). Training the neural network with cytometry image data from the same cell enabled researchers to template the signals quickly and accurately, thereby quantifying the cell’s deformability. However, because apoptotic cells generally exhibit lower deformability and a broad size distribution—both of which amplify variability in deformability measurements—the researchers used an impedance phase metric to gate only live cell events, thereby excluding apoptotic cells from the analysis. These studies demonstrated MLP’s dual capability in both engineering design automation (through precise geometric-performance mapping) and biophysical analysis (via multimodal signal interpretation), with quantitative validation underscoring its reliability in cell analysis.

MLP leverages multilayer nonlinear transformations to learn intricate features with a strong expressive ability and intuitive training processes, and it is particularly effective for small-scale datasets. While fully connected architecture requires massive parameters and high computational complexity, and when processing structured data like cellular images, the flattening of spatial inputs into 1D vectors erodes structural information while incurring prohibitive parameterization (leading to computational complexity), ultimately compromising generalization capabilities and triggering overfitting risks easily. In contrast, a CNN fundamentally reduces these restrictions through localized feature extraction and spatial hierarchy preservation, achieving substantial reductions in parameter count and computational overhead while enabling parallel processing acceleration. This architectural superiority positions the CNN as a computationally efficient solution for large-scale image analysis tasks requiring structural awareness.

### Convolutional neural network

The CNN excels in processing high-dimensional data such as images and videos, with core architectural components including convolutional layers, pooling layers, and fully connected layers (*172*). Distinguished by their hierarchical feature extraction capabilities and computational efficiency, the CNN surpasses traditional ML methods in handling complex visual data, establishing them as the preferred architecture for vision-centric DL tasks. On the basis of algorithm functionality and diverse objectives in visual pattern analysis, the CNN could be categorized in this section into the classical CNN and the YOLO framework.

### Classical CNN architectures in cell sorting applications

CNN-based architectures have been implemented in label-free detection modalities, such as backscattering FCM and holographic microscopy, notably reducing dependence on fluorescence labeling while enhancing operational accessibility and biological compatibility (*173*, *174*).

Classical CNN architectures leverage hierarchical feature extraction through convolutional kernels with local receptive fields and weight-sharing mechanisms, enabling efficient multiscale pattern recognition. This intrinsic capability endows CNNs with superior generalization performance and computational efficiency in cell analysis, particularly in scenarios of cell sorting that demand high-throughput processing of morphologically complex biological samples. Vora *et al.* (*175*) enhanced the detection of CTCCs by combining confocal backscatter FCM with an ensemble of 10 CNN models, achieving a false positive rate of 0.78 events per minute and a Pearson correlation coefficient of 0.943 for target events (Fig. 7B). Their system maintained 72% purity and 35.3% sensitivity in whole-blood analyses, even for rare CTCC populations as small as two cells. Gangadhar *et al.* (*176*) designed a label-free detection method and holographic microscopy. This research developed a custom s-Net model on the basis of optimizing the CNN model and comparing it with models such as ResNet50. The method achieved high levels of accuracy, sensitivity, and specificity while notably reducing training and testing times. However, this method of extracting features to distinguish cells requires a priori knowledge and features of cells and may not be applicable to other types of cells. Karar *et al.* (*177*) combined 1D CNN with long short-term memory networks to automate pancreatic ductal adenocarcinoma (PDAC) diagnosis through urinary biomarker analysis, attaining 97% diagnostic accuracy. Moradi *et al.* (*178*) implemented a cost-effective microfluidic-epifluorescence system coupled with a custom CNN, achieving a 98.57% overall accuracy in leukocyte subtyping, with subtype-specific accuracies exceeding 99% for lymphocytes, monocytes, eosinophils, and basophils, while outperforming VGG16 and ResNet18 in processing speed for real-time applications. To solve the problem of data imbalance, a weighted class loss function was used, and the *K*-fold cross-validation method was used to improve accuracy. Herbig *et al.* (*179*) optimized single-cell isolation using serpentine microchannels and CNN-based image analysis, achieving a 97.4% test accuracy in distinguishing singlets from doublets. These studies collectively validate CNN’s capacity to balance analytical precision with computational efficiency across heterogeneous biological detection paradigms. The CNN is the preferred architecture for image classification tasks, but the CNN usually requires more computing time.

Classical CNN architectures demonstrate robust feature extraction capabilities and spatial invariance through hierarchical architectures that efficiently capture multiscale cellular image patterns, with their parameter-sharing mechanism significantly reducing computational complexity. However, the reliance on localized receptive fields limits global contextual awareness, often leading to feature degradation in multiscale detection scenarios, particularly for small-cell targets where critical morphological details may be lost during the progressive downsampling process. YOLO frameworks address these limitations by integrating global contextual analysis with multiscale feature fusion strategies, enabling simultaneous detection of cellular structures across spatial hierarchies. By aggregating low-resolution semantic features and high-resolution texture details, this approach effectively enhances sensitivity to subcellular features in small target cells while preserving computational efficiency.

### YOLO framework in cell sorting applications

The YOLO framework enhances detection speed by transforming object detection into an end-to-end regression problem, enabling simultaneous prediction of multiple cellular targets’ class identities and bounding box coordinates through the single forward propagation. This architectural innovation achieves real-time detection speeds, which is critical for high-throughput cell sorting systems (*180*). On the basis of CNN’s hierarchical feature extraction capabilities, the YOLO framework also introduces specialized detection heads and composite loss functions that synergistically optimize classification accuracy and spatial localization precision (*181*).

YOLO-based frameworks have demonstrated exceptional performance across diverse cell sorting platforms through architectural innovations tailored for biomedical imaging. Guo *et al.* (*182*) implemented YOLOv8 on an active matrix digital microfluidics, achieving a 98.5% sorting accuracy with a 96.49% purity and 80% recovery rates in HeLa cell and polystyrene bead classification (Fig. 7C). To enhance the robustness of the model, a camera was used to capture images of red blood cell samples at three different focal planes (±10 μm). Yang *et al.* (*183*) trained the sample images with a YOLOv4 model, adjusting the model parameters to identify all cells, resulting in an average recognition accuracy of 89.24%. Du *et al.* (*184*) combined YOLOv4 with bright-field image cytometry, reporting average precisions (APs) of 98.63% (HT29), 99.04% (A549), and 98.95% (KYSE30) for cancer cell detection. They also proposed a multiframe correlation analysis algorithm to optimize single-frame detection results, further improving the APs of HT29, A549, and KYSE30 to 99.40, 99.52, and 99.47%, respectively. Gardner *et al.* (*185*) leveraged YOLOv5 for single-cell resolution within microfluidic droplets, achieving an 11% accuracy improvement over prior detection systems. These implementations collectively validate YOLO’s capacity to balance real-time processing speeds with subcellular detection fidelity across heterogeneous biological samples.

The YOLO framework achieves efficient object detection through its single-stage architecture, which simultaneously performs target localization and classification within a single forward propagation, enabling real-time processing speeds critical for high-throughput systems. By leveraging the global image context for prediction, the YOLO framework notably reduces the false positive rate of background while maintaining computational efficiency through parameter-optimized detection heads and composite loss functions. The streamlined architecture facilitates rapid optimization and deployment across diverse platforms, making it particularly suited for real-time applications requiring robust detection fidelity in dynamic cellular environments.

### Residual networks

ResNet introduces a residual learning mechanism and skips connections to allow gradients to be propagated directly to the shallow layers, thus addressing the issues of gradient vanishing and network degradation in deep network training, showing outstanding performance in image-based cell object detection and classification (Fig. 7D) (*186*). ResNet’s residual architecture has demonstrated exceptional performance in cellular analysis through multiple implementations. Vanhoucke *et al.* (*187*) used ResNet50 to classify droplet-encapsulated single cells and multicellular aggregates, achieving accuracy improvements from 75.9 to 91.7% for single-cell droplets and from 86.9 to 93.4% for multicellular clusters, showcasing robust feature extraction in complex droplet images. Simultaneously, Hashemzadeh *et al.* (*188*) used ResNet18 to classify lung cancer cell lines via microfluidic-cultured samples, attaining a 99.77% mean accuracy in distinguishing malignant cells from normal cells. Lee *et al.* (*115*) used the ResNet18 model to achieve fast processing on cell classification. The model achieved sorting purities of 98.0, 95.1, and 94.2% for 15- and 10-μm beads, HL-60 and Jurkat cells, and HL-60 and K562 cells, respectively, with a throughput of up to 82.8 events per second. These studies collectively highlight ResNet’s capacity to balance depth-induced representational power with operational efficiency, particularly in scenarios requiring micrometer-scale morphological discrimination under dynamic fluidic conditions. Jeon *et al.* (*189*) used a multidimensional dual-spiral microfluidic device combined with an improved ResNet152 model to process bright-field images, achieving efficient leukocyte sorting. The red blood cell removal rate reached ~99.999%, with a leukocyte recovery rate of around 80%. Ultimately, the leukocyte purity attained 95%, and the average accuracy in the three-class classification task was 81.4%.

ResNet effectively addresses depth-related limitations and stabilizes gradient flow in deep neural networks through skip connections, and its modular architecture notably enhances hierarchical feature extraction capabilities. ResNet enables the capture of the high-level semantic features from microfluidic cellular images and the subtle morphological variations in cell shape, size, and texture. This structural innovation facilitates the progressive refinement of discriminative cellular signatures across network depths, ultimately enabling high-precision classification for phenotypically similar cell populations.

DL-based microfluidic cell sorting systems can learn complex features directly from large-scale cell images. This eliminates the need for manually defined indicators. By capturing subtle yet critical information, such as edge textures and internal structures, these systems achieve recognition and sorting performance comparable to, or surpassing, manual assessment. For example, systems have been applied successfully to distinguishing WBC subtypes.

Nevertheless, several limitations remain. First, deep models are highly dependent on the volume and diversity of training data. When datasets are insufficient or exhibit limited heterogeneity, models are prone to overfitting, which reduces their adaptability to new environments, such as varying microscopic imaging conditions or chip materials. Moreover, deep models remain sensitive to input noise and perturbations; even minor defocus blur or illumination fluctuations can notably affect outputs. To enhance stability, some approaches capture images at multiple focal planes, underscoring that current models still lack robustness. Last, the training and inference of deep networks require substantially greater computational resources compared to traditional ML, resulting in high computational costs and time-consuming processes. This presents a major barrier to the deployment of microfluidic systems in portable, real-time testing scenarios.

As introduced in this chapter, traditional ML methods rely on handcrafted feature extraction, and it uses simple methods to handle with relatively simple tasks. However, their generalization ability is limited when dealing with complex cell structures and high-dimensional data. DL methods, represented by neural networks (such as classic CNN, YOLO, ResNet, etc.), can learn complex local and global features from cell data without manual intervention, enhancing the accuracy and generalization ability of cell classification. These methods continue to evolve; for example, the third-generation neural network, a spiking neural network, offers advantages in efficiently processing sparse asynchronous event data and bioinspired spatiotemporal feature extraction. He *et al.* (*190*) used a multiobject tracking-spiking neural network pipeline within their neuromorphology-driven video-activated cell sorter framework, capturing rich temporal and spatial information in asynchronous and sparse-driven lightweight computation, which led to a significant enhancement in the performance of cell classification tasks under complex scenarios. This study offered a previously unidentified technological paradigm for the development of efficient and precise intelligent bioinformatics processing systems.

## CHALLENGES AND FUTURE PERSPECTIVES

The deep integration of microfluidic technologies with ML is redefining the scientific and technological boundaries of cell sorting. Current advancements synergize physical field–based sorting mechanisms—including inertial forces, DEP, and acoustofluidics—with DL algorithms such as MLP and CNN, enabling the multidimensional analysis of cellular morphology, motion trajectories, and multimodal biophysical signatures. This interdisciplinary collaboration not only overcomes the limitations of single-field actuation but also provides dynamic data support for single-cell sequencing and precision medicine with real-time feature visualization and high-throughput processing capabilities. In complex biological samples such as CTCs and exosomes, the coordinated optimization of algorithm-driven feature extraction and physical field manipulation has notably enhanced the efficiency and specificity of rare cell isolation. Future advancements in ML-enhanced microfluidic sorting may focus on the following directions.

### Innovations in microfluidic system design

Future microfluidic platforms will evolve toward functional integration and multidimensional manipulation. Integrated systems combining sorting modules with downstream in situ detection and single-cell analysis units could establish closed-loop diagnostic platforms with “sample-to-answer” functionality. For instance, cascaded designs coupling inertial sorting with nanosensing technology may enable rare cell isolation and target biomarker analysis simultaneously. In addition, programmable multiphysical field coupling (e.g., optomagnetoacoustic hybrid actuation) will support label-free subpopulation sorting by dynamically matching cellular heterogeneity. The convergence of microfluidics and optofluidics could further promote 3D single-cell manipulation and organoid construction, expanding new possibilities in regenerative medicine.

### Advancements in machine learning algorithms

As for ML, morphological characteristics of cells—including size, shape, and motion trajectories—can be further extracted and identified. Semantic segmentation models may address cell aggregation challenges to improve the distinguishing ability of the mode. By constructing multimodal datasets incorporating video stream, algorithms can adapt to identification requirements under diverse experimental conditions. By leveraging fine-tuned large language models (LLMs; e.g., GPT and LLaMA) and multimodal fusion techniques, the intelligent closed-loop systems could be developed to enable real-time cell recognition followed by adaptive optimization of microfluidic parameters (flow rate, channel geometry, etc.). This dynamic feedback mechanism would enhance sorting stability and efficiency under fluctuating cellular densities, ensuring high purity and throughput.

Microfluidic cell sorting enabled by ML demonstrates higher precision, automation, and throughput. However, it should be noted that the deep integration of microfluidics and ML still faces significant difficulties and challenges. From a technical perspective, the field of microfluidic sorting has undergone a transition from traditional digital image processing to deep neural network frameworks. DL methods, primarily based on CNNs and their variants (such as the image-activated cell sorting pipeline and various CNN-based detectors/segmenters), have achieved relatively mature engineering feasibility in closed-loop online sorting with millisecond-level decision making. It is noteworthy that in biomedical imaging and cellular microscopy scenarios, vision transformers have gradually demonstrated advantages in both performance and interpretability for tasks such as cell segmentation, classification, and phenotypic characterization. Furthermore, lightweight or self-supervised variants of vision transformers have emerged, designed for applications such as microscopic cell segmentation/classification, weakly supervised pathology tasks, and foundation models tailored for microscopy. Nevertheless, most of these advances remain limited to offline inference or nonmicrofluidic real-time conditions (*191*). In addition, current research focuses on image/video modalities, with insufficient attention paid to text modalities. As a result, research on the application of LLMs is also clearly insufficient: Although some digital microfluidic work has proposed using LLMs to assist semantically driven experimental control, process orchestration, and human-computer interaction or demonstrated a proof of concept of “LLM + target detection” for automated biological processes (*192*, *193*), systematic frameworks and public examples for closed-loop control, cross-device command docking, and safety and compliance modules for microfluidic cell sorting are still scarce, and reproducible evaluation and compliance paths have not yet been formed.

This review highlights recent progress in merging microfluidic cell sorting with ML, underscoring their synergistic potential in large-scale biomedical research. Continued innovation and interdisciplinary convergence in this field are poised to drive breakthroughs in disease diagnostics, drug screening, and personalized therapeutics.
